# Coinfection with *Leishmania major* and *Staphylococcus aureus* enhances the pathologic responses to both microbes through a pathway involving IL-17A

**DOI:** 10.1371/journal.pntd.0007247

**Published:** 2019-05-20

**Authors:** Tiffany Y. Borbón, Breanna M. Scorza, Gwendolyn M. Clay, Fellipe Lima Nobre de Queiroz, Alan J. Sariol, Jayden L. Bowen, Yani Chen, Bayan Zhanbolat, Corey P. Parlet, Diogo G. Valadares, Suzanne L. Cassel, William M. Nauseef, Alexander R. Horswill, Fayyaz S. Sutterwala, Mary E. Wilson

**Affiliations:** 1 Department of Microbiology and Immunology, University of Iowa, Iowa City, IA, United States of America; 2 Medical Scientist Training Program and the Carver College of Medicine, University of Iowa, Iowa City, IA, United States of America; 3 Interdisciplinary Ph.D. Program in Immunology, University of Iowa, Iowa City, IA, United States of America; 4 Interdisciplinary Ph.D. Program in Molecular Medicine, University of Iowa, Iowa City, IA, United States of America; 5 Department of Internal Medicine, University of Iowa, Iowa City, Iowa City, IA, United States of America; 6 Iowa Inflammation Program, Department of Internal Medicine, University of Iowa, Iowa City, IA, United States of America; 7 Veterans’ Affairs Medical Center, Iowa City, IA, United States of America; 8 Conselho Nacional de Desenvolvimento Cientifico e Tecnológico (CNPq), Brasilia, Brazil; 9 Department of Medicine, Cedars-Sinai Medical Center, Los Angeles, CA, United States of America; 10 Department of Immunology and Microbiology, University of Colorado Denver—Anschutz Medical Campus, Aurora, CO, United States of America; Instituto de Ciências Biológicas, Universidade Federal de Minas Gerais, BRAZIL

## Abstract

Cutaneous leishmaniasis (CL) is a parasitic disease causing chronic, ulcerating skin lesions. Most humans infected with the causative *Leishmania* protozoa are asymptomatic. *Leishmania* spp. are usually introduced by sand flies into the dermis of mammalian hosts in the presence of bacteria from either the host skin, sand fly gut or both. We hypothesized that bacteria at the dermal inoculation site of *Leishmania major* will influence the severity of infection that ensues. A C57BL/6 mouse ear model of single or coinfection with *Leishmania major*, *Staphylococcus aureus*, or both showed that single pathogen infections caused localized lesions that peaked after 2–3 days for *S*. *aureus* and 3 weeks for *L*. *major* infection, but that coinfection produced lesions that were two-fold larger than single infection throughout 4 weeks after coinfection. Coinfection increased *S*. *aureus* burdens over 7 days, whereas *L*. *major* burdens (3, 7, 28 days) were the same in singly and coinfected ears. Inflammatory lesions throughout the first 4 weeks of coinfection had more neutrophils than did singly infected lesions, and the recruited neutrophils from early (day 1) lesions had similar phagocytic and NADPH oxidase capacities. However, most neutrophils were apoptotic, and transcription of immunomodulatory genes that promote efferocytosis was not upregulated, suggesting that the increased numbers of neutrophils may, in part, reflect defective clearance and resolution of the inflammatory response. In addition, the presence of more IL-17A-producing γδ and non-γδ T cells in early lesions (1–7 days), and *L*. *major* antigen-responsive Th17 cells after 28 days of coinfection, with a corresponding increase in IL-1β, may recruit more naïve neutrophils into the inflammatory site. Neutralization studies suggest that IL-17A contributed to an enhanced inflammatory response, whereas IL-1β has an important role in controlling bacterial replication. Taken together, these data suggest that coinfection of *L*. *major* infection with *S*. *aureus* exacerbates disease, both by promoting more inflammation and neutrophil recruitment and by increasing neutrophil apoptosis and delaying resolution of the inflammatory response. These data illustrate the profound impact that coinfecting microorganisms can exert on inflammatory lesion pathology and host adaptive immune responses.

## Author summary

Cutaneous leishmaniasis (CL) is a vector-borne ulcerating skin disease affecting several million people worldwide. The causative *Leishmania* spp. protozoa are transmitted by infected phlebotomine sand flies. During a sand fly bite, bacteria can be coincidentally inoculated into the dermis with the parasite. *Staphylococcus aureus* is the most common bacterium in CL skin lesions. Symptomatic CL is characterized by papulonodular skin lesions that ulcerate and resolve with scarring, although most cutaneous *Leishmania* infections are asymptomatic. We sought to explore factors that determine whether infection with a cutaneous *Leishmania* species would result in symptomatic CL rather than asymptomatic infection. We hypothesized that local bacteria promote the development of symptomatic CL lesions during infection with *Leishmania major*. We discovered that cutaneous lesions were significantly larger in mice inoculated simultaneously with *S*. *aureus* and *L*. *major* than in mice infected with either organism alone. Coinfection led to increased *S*. *aureus* growth in skin lesions, whereas *L*. *major* parasite numbers were unchanged by coinfection. The size of the exacerbated lesion correlated with early increased numbers of neutrophils and elevated levels of proinflammatory cytokines IL-1β and IL-17A during the first 7 days, and with sustained increases in IL-17A through 28 days of coinfection. Neutralizing antibody experiments suggested IL-17A was partially responsible for lesion exacerbation during coinfection, whereas IL-1β was important for both control of early lesion exacerbation and promotion of IL-17A production. These data suggest that treatment of symptomatic CL targeting the parasite, local commensal bacteria, and host proinflammatory IL-17A immune responses might improve the outcome of CL.

## Introduction

Leishmaniasis constitutes a spectrum of diseases with distinct clinical forms usually caused by different species of *Leishmania* protozoa [1]. Each of the *Leishmania* species can also lead to highly variable clinical outcomes in different individuals, ranging from asymptomatic to severe infection. The most common disease form is cutaneous leishmaniasis (CL), which presents as lesions that often ulcerate and usually spontaneously resolve within weeks to months [2]. *L*. *major* is a species responsible for a substantial portion of the CL burden in the Eastern Hemisphere [3].

An underappreciated variable influencing CL disease outcome is the local microbial flora at the site of mammalian infection [4–8]. However, specific interactions between *Leishmania* parasites, local bacteria, and the host immune response are underexplored. *Leishmania* spp. are usually introduced into a susceptible mammalian host through the bite of an infected female phlebotomine sand fly. The insect bites by repeated probing activity, a behavior that amplifies in flies harboring *Leishmania* spp. parasites, forming a pool of blood in the dermis into which the parasite is inoculated [9–11]. In addition to commensal bacteria on the host’s skin, recent data show the infection site is also exposed to microbes from the sand fly gut [12]. Ulcerated lesions provide a portal for bacterial invasion, occasionally leading to superinfection [13–15]. *Staphylococcus* and *Streptococcus* species are the two most common bacterial genera that have been detected in surveys of CL lesion microbiota [13–15]. Sand fly midgut microbiota include bacterial species belonging to the families *Staphylococcaceae* and *Streptococcaceae* within the phylum *Firmicutes* [16, 17].

Bacteria egested by sand flies into the skin during a blood meal can also incite an inflammatory response [12]. *Staphylococcus aureus* infection is known to activate the NLRP3 inflammasome, a multi-protein complex that activates caspase-1 which in turn cleaves and prompts release of IL-1β and IL-18 [18–20]. Inflammasomes are activated during human CL caused by *L*. *braziliensis*, and local IL-1β exacerbates lesion pathology in murine models of CL [21–25]. IL-1β also plays a role during infections with visceralizing species of *Leishmania* [12, 26–28].

Based on the above observations, we hypothesized that bacteria coinfecting the site of *L*. *major* infection will activate proinflammatory mediators and thereby modify the host response to *L*. *major* infection. Our data showed that *S*. *aureus* coinfection indeed had a profound influence on the outcome of *L*. *major* lesions, leading to lesion exacerbation within the first four weeks of coinfection.

## Materials and methods

### Ethics statement

All experiments with vertebrate animals were performed in accordance with recommendations in the Guide for the Care and Use of Laboratory Animals and were approved by the Institutional Animal Care and Use Committees (IACUC) of the University of Iowa (protocol 7071099) and the Iowa City Veterans’ Affairs Medical Center (ACORP protocol 1690501). All procedures, including anesthesia and experimental endpoints, were performed in accordance with American Veterinary Medicine Association (AVMA) guidelines, and were approved by review committees at the University of Iowa and the Iowa City VA Medical Center.

### Mice

Four- to six-week-old C57BL/6N female mice purchased from Charles River were used in the experiments in this study. Mice were housed under specific pathogen-free conditions at the Iowa City Veteran’s Affairs Medical Center Animal Research Facility.

### Microbial culture and preparation

Procedures for *L*. *major* and *S*. *aureus* preparation and co-inoculation by intradermal injection into mice ears are described in the protocol available online: dx.doi.org/10.17504/protocols.io.vdse26e. *L*. *major* IA-2 strain was recently isolated from a patient who acquired CL in Iran. The promastigote forms of either wild-type *L*. *major* IA-2 or IA-2 expressing genes encoding luciferase and mCherry were grown at 26°C in Schneider’s *Drosophila* medium + *L*-glutamine (Gibco by Life Technologies) supplemented with 10% heat-inactivated fetal bovine serum (FBS) (SAFC Industries), 2 mM *L*-glutamine (Gibco by Life Technologies), 50 μg/mL gentamicin sulfate (IBI Scientific), and 1.2 μg/mL biopterin (Cayman Chemical Company). Metacyclic promastigotes were isolated by Ficoll-Paque PLUS (GE Healthcare) density gradient as previously described [29] and suspended to a concentration of 10^6^ parasites in 10 μL of phosphate buffered saline (PBS; Gibco by Life Technologies). For experimental conditions, parasite suspensions were mixed with liquid media containing buffer along or *S*. *aureus* immediately before inoculation into mice.

Newman is a methicillin-sensitive *S*. *aureus* [MSSA; α-toxin (*hla)* positive] strain [30]. *S*. *aureus* LAC::*lux* and *S*. *aureus* LAC pCM29 (chloramphenicol resistant) are methicillin-resistant USA300 strains that express bacterial luciferase or green fluorescent protein (GFP), respectively [MRSA, *hla+*] [31–33]. *S*. *aureus* MNPE [*hla*+, toxic shock syndrome superantigen (TSST-1) positive] is a USA200 strain, which was kindly provided by Dr. Patrick Schlievert of the University of Iowa [34]. Bacteria were grown at 37°C on a semisolid tryptic soy agar (TSA) plate overnight. Single colonies were cultured in tryptic soy broth (TSB) at 37°C with shaking overnight. Overnight cultures were diluted 1:100 in TSB and grown to an optical density (OD_600_) of 0.5. Ten mL of bacterial cultures were washed by centrifugation in PBS. Based on the estimate that an OD_600_ of 1.0 corresponds to 3x10^5^ colony-forming units (CFU) per μL, 10^4^ CFUs were suspended in 10 μL PBS either with or without *L*. *major*. Bacterial CFUs in the final injection doses were confirmed by serial dilutions on TSA plates incubated overnight at 37°C.

### Intradermal infections

Mice were anesthetized by intraperitoneal (i.p.) injection of ketamine (80 mg/kg, Ketalar, Par Pharmaceutical Cos., Inc.) and xylazine (10 mg/kg, AnaSed, LLOYD Laboratories, LLOYD Inc.), and then intradermally injected in one ear pinna with 10^6^ *L*. *major* parasites, 10^4^ CFUs of *S*. *aureus*, or a mixture of both in 10 μL volume. Lesion dimensions were measured daily for one week followed by weekly measurements for three additional weeks. Lesion measurements were made using a Mitutoyo Flat Anvil Dial Thickness Gage (0–22 mm) in 0.01 mm increments for thickness, and a ruler for length and width. Lesion volume was calculated using the formula for volume of an ellipsoid: $lesion\;volume=\frac{4}{3}\times \pi \times \frac{length}{2}\times \frac{width}{2}\times \frac{thickness}{2}$. For the five-day *S*. *aureus* preliminary dosage experiment (S1 Fig), lesion area was calculated using the formula for the area of an ellipse: $lesion\;area=\pi \times \frac{length}{2}\times \frac{width}{2}$. At experiment endpoints, mice were euthanized in accordance with AVMA guidelines as approved by the University of Iowa IACUC.

### *In vivo* imaging of cutaneous bacterial burden

After intradermal inoculation of 10^4^ CFUs of *S*. *aureus* LAC::*lux*, expressing the *Photorhabdus luminescence lux* operon. Mice were imaged under anesthesia by inhalation of 2% isoflurane (Piramal Enterprises Limited). Photons, which are emitted only from live, luminescent bacteria, were quantified during a 1-minute exposure using the IVIS-200 (*in vivo* imaging system) and Living Image software from Xenogen. Total light emissions (flux) in a uniformly defined circular region of interest over the ear infection site were quantified at different time points of infection in each mouse.

### Ear histology and DNA and RNA extraction

Ears were harvested at four weeks post-injection. Active lesions were bisected, and a portion was paraffin-embedded. Three μm sections were cut and stained with hematoxylin and eosin and analyzed using a semi-quantitative histological scoring method [35]. The remaining ear tissue was homogenized. Portions were either suspended in Cell Lysis Solution (QIAGEN Sciences) and DNA was extracted using the Puregene DNA isolation Kit (Gentra) protocol for mouse tail snips or suspended in 1 mL of TRIzol Reagent (Invitrogen) and RNA was extracted using R.Z.N.A Total RNA Kit I (Omega Bio-Tek R6834-02). Lysostaphin (0.01 U/mL, AMBI Products, LLC) was added to DNA extraction buffer for experiments requiring *S*. *aureus* quantification by qPCR.

### Microbial quantification by qPCR and bacterial colony-forming units

Quantitative polymerase chain reaction (qPCR) was performed on DNA extracted from tissues of infected or uninfected (control) mice using kinetoplastid DNA (kDNA5) primers as previously described [36]. Parasite genome equivalents were calculated based on a standard curve with varied amounts of *L*. *major* promastigote DNA in a constant amount of mouse ear DNA. *S*. *aureus* relative genome equivalents were determined using primers and probe specific to the thermonuclease *nuc* gene, and comparison to a standard curve of *S*. *aureus* DNA diluted in mouse ear DNA [37]. Quantitative PCR reactions were performed in 96-well fast plates in an Applied Biosystems QuantStudio 7 Flex Real-Time PCR System (Life Technologies). Preliminary experiments documented that the qPCR method correlates with bacterial counts. To verify live *S*. *aureus* counts, ears were homogenized in 0.5 mL of PBS and homogenized using a Tissue Master 125 (OMNI International). 10 μL of ear homogenate was spread onto TSA plates, incubated at 37°C overnight, colony-forming units (CFUs) were counted, and the total number of live *S*. *aureus* bacteria was calculated.

### Gene expression assays

Reverse transcriptase-quantitative polymerase chain reaction (RT-qPCR) was performed on RNA obtained from harvested ear skin. Complementary DNA (cDNA) was generated using the Superscript III Reverse Transcriptase First Strand Synthesis System (Invitrogen) using the manufacturer’s protocol with random hexamers. Samples were pre-amplified using pooled Taqman assays (ABI) and PreAmp Master Mix (Fluidigm) according to the Gene Expression Preamplification with Fluidigm PreAmp Master Mix and TaqMan Assays protocol (Fluidigm). Expression of 48 control and inflammatory genes was assessed using a 48x48 qPCR dynamic array (Fluidigm), with a panel of Taqman assays (ABI), according to manufacturer’s instructions. Data were calculated by the ΔΔC_T_ method, using either GAPDH or GUSB in each experiment to normalize transcripts within samples. Each normalized ΔC_T_ value was compared to the average ΔC_T_ for the same transcript in sham injected mice to get the -ΔΔC_T_, yielding the log_2_(fold change).

### Lymph node cell stimulation and multiplex cytokine assays

The submandibular lymph nodes draining ear lesions were homogenized in 200 μl of RP10 media, which consists of 450 mL RPMI 1640 (Gibco by Life Technologies), 50 mL FBS, 1 mM *L*-glutamine (Gibco by Life Technologies), 100 units/mL penicillin/streptomycin (Gibco by Life Technologies) in 1.5 μL microfuge tubes mini-pestles. One times 10^5^ viable lymph node cells in 100 μL were transferred to each well of a 96-well round-bottom plate (COSTAR). Cells were incubated for 72 hours at 37°C, 5% CO_2_ with 3x10^5^ *L*. *major* IA-2 live promastigotes as a source of live parasite antigen. After 48 hours, supernatants were collected and stored at -80°C. Cytokines were quantified using multiplex fluorescent bead arrays for murine IL-2, IL-4, IL-5, IL-10, IL-12(p70), IL-17A, IFNγ, and TNFα from (Biorad) on a Luminex 200 detection instrument (Luminex Corporation).

### Surface marker and intracellular cytokine staining for flow cytometry

Single cell suspensions of dermal cells were obtained at the indicated times post-infection (p.i.) from mouse ears by separating dermal sheets and incubating dermis-side down in 0.5 mL of 0.2 mg/mL of Liberase DL (Roche) in RP10 medium in a 24-well plate for 1 hour at 37°C, 5% CO_2_, agitating every 15 minutes. One μL of Benzonase Nuclease, Purity >99% (EMD Millipore) was added during the last 15 minutes. Skin cells were homogenized by passage through a 70 μm nylon cell strainer, centrifuged at 3000 rpm for 10 minutes, and resuspended in RP10. Single cell suspensions from draining submandibular lymph nodes were obtained as described above for lymph node cell stimulation and multiplex assays. Total cell number was determined by counting cells in 10 μL of each sample on a hemocytometer.

Cells were washed three times in FACS buffer [PBS, 2 μM EDTA (Fisher Scientific), 1% v/v FBS (SAFC Industries) and 0.1% w/v sodium azide (Sigma-Aldrich)] and suspended in antibodies to surface markers diluted 1:400 in FACS buffer. Cells stained for surface markers were washed by centrifugation, fixed in PBS supplemented with 2% paraformaldehyde (Sigma-Aldrich) (fixation buffer), and stored protected from light at 4°C overnight.

Cells harvested for intracellular cytokine staining (ICS) were cultured for 4–5 hours in RP10 with 2 μg/mL of brefeldin A (eBiosciences), 0.1 μg/mL of phorbol 12-myristate 13-acetate (PMA; Sigma-Aldrich), and 1 μg/mL of ionomycin (Sigma-Aldrich) at 37°C, 5% CO_2._ Cells were suspended in Permeabilization Buffer (0.1% saponin, 0.09% sodium azide, eBioscience) with 1:200 dilutions of intracellular anti-cytokine antibodies at 4°C for 20–30 minutes. Cells were resuspended in fixation buffer at 4°C until analysis.

For IL-17A ICS of CD11b^+^ cells, stimulated cells were resuspended in TruStain FcX anti-mouse CD16/32 antibody (Biolegend) at 1.0 μg/well in 50 μL for 10 minutes on ice prior to immunostaining. Cells were surface stained and permeabilized as described above, and half of each sample was stained for intracellular IL-17A with 1:200 dilutions of PE anti-IL17A clone TC11-18H10.1 (Biolegend). The other half of each sample was stained with isotype control PE rat IgG1 kappa (Biolegend). Cells were resuspended in fixation buffer at 4°C until analysis.

Fluorescent anti-mouse antibodies used for surface staining: PE-Cy7 anti-CD4 clone GK1.5, and FITC anti-CD8a clone 53–6.7 from eBioscience; Brilliant Violet 421 anti-CD45 clone 30-F11, APC-Cy7 anti-CD11b clone M1/70, Alexa Fluorophore 700 anti-CD90.2 (Thy1.2) clone 30-H12, FITC anti-TCR γ/δ clone GL3, APC anti-Ly6G clone 1A8, and FITC anti-Ly6C clone HK1.4 from BioLegend; PerCP-Cy5.5 anti-CD11b M1/70 from BD Bioscience. Intracellular cytokine stains were PE-Cy7 anti-IFNγ clone XMG1.2, APC anti-pro-IL-1β clone NJTEN3 from eBioscience, and PE anti-IL17A clone TC11-18H10.1 from Biolegend. Flow cytometry was performed using the Becton Dickinson LSR II with 405 nm, 488 nm, 561 nm, and 639 nm lasers using BD FACSDiva (BD Biosciences) software. ICS gates were determined using fluorescence minus one (FMO) controls. Percentage of IL-17A^+^ CD11b^+^ cells was determined by subtracting the percent of PE^+^ cells in the IgG1 isotype controls from the percent of PE IL-17A^+^ cells, and the adjusted percentage was then multiplied by the total number of cells in that sample. Data were analyzed using FlowJo Software.

### IL-1β enzyme-linked immunosorbent assay (ELISA)

Ears from 3 days p.i. were snap frozen in 1.5 mL microcentrifuge tubes in liquid nitrogen and stored at -80°C. Frozen ears were transferred to 5 mL round bottom polystyrene tubes in 300 μL of Cell/Tissue Extraction Buffer [100 mM Tris, pH 7.4 (RPI Corp.), 150 mM NaCl (RPI Corp.), 1 mM EGTA (Fisher Scientific), 1 mM EDTA (Fisher Scientific), 1% Triton X-100 (Sigma), 0.5% sodium deoxycholate (Sigma-Aldrich)] with 1 mM protease inhibitor cocktail (Roche). Ears were homogenized using a Tissue Master 125 (OMNI International) and agitated on a rotating platform for 2 hours at 4°C. Tissue homogenates were microcentrifuged for 20 minutes at 13,000 rpm at 4°C and the supernatants were transferred into clean 1.5 mL microcentrifuge tubes and stored at -80°C. A murine IL-1β ELISA (R&D Systems) was performed on collected supernatants from homogenized ears and read using the FLUOstar Omega plate reader (BMG Labtech).

### Neutrophil NADPH oxidase and apoptosis assays

Single cells suspensions were obtained one day p.i. from mouse ears as described above. Cells were surface stained with Brilliant Violet 711 anti-Ly6G, Brilliant Violet 421 anti-CD45 clone 30-F11, and APC-Cy7 anti-CD11b clone M1/70 from Biolegend. To assess the ability of neutrophils to activate the phagocyte NADPH oxidase, cells were incubated in PBS with 100 ng/mL of PMA (Sigma Aldrich) and 10 μM dihydrorhodamine 123 (DHR123, Sigma Aldrich) for 15 minutes at 37°C, 5% CO_2_, and then washed in PBS and analyzed by flow cytometry. To assay for apoptosis, cell suspensions were resuspended in 100 μL Annexin V binding buffer (Biolegend) and incubated with 5 μL of APC Annexin V (Biolegend) for 10–15 min at room temperature in the dark. Within 10 minutes prior to analysis by flow cytometry, 10 μL of Propidium Iodide Staining Solution (Biolegend) was added to each sample.

### Antibody neutralizations

Female C57BL/6 mice were injected intraperitoneally with 0.5 mg anti-mouse IL-17A clone 17F3 antibody (Bio X Cell *InVivo*Mab) or isotype control (*InVivo*MAb mouse IgG1 clone MOPC-21). Other infected mice were injected with 0.5 mg anti-IL-1β antibody (Bio X Cell *InVivo*MAb anti-mouse/rat IL-1β clone B122) or isotype control (*InVivo*MAb polyclonal Armenian hamster IgG) in PBS every 3 days starting one day prior to intradermal ear infection, for a total of 9 days. Lesion size was monitored as described above.

### Statistical analyses

Statistical analyses were performed using GraphPad Prism software. Data were analyzed by one-way or two-way ANOVA with Tukey’s post-test for multiple comparisons, or by student’s *t*-test.

## Results

### Time- and location-based effects of coinfecting microbes on host response to *S*. *aureus* and *L*. *major*

**Fig 1:**
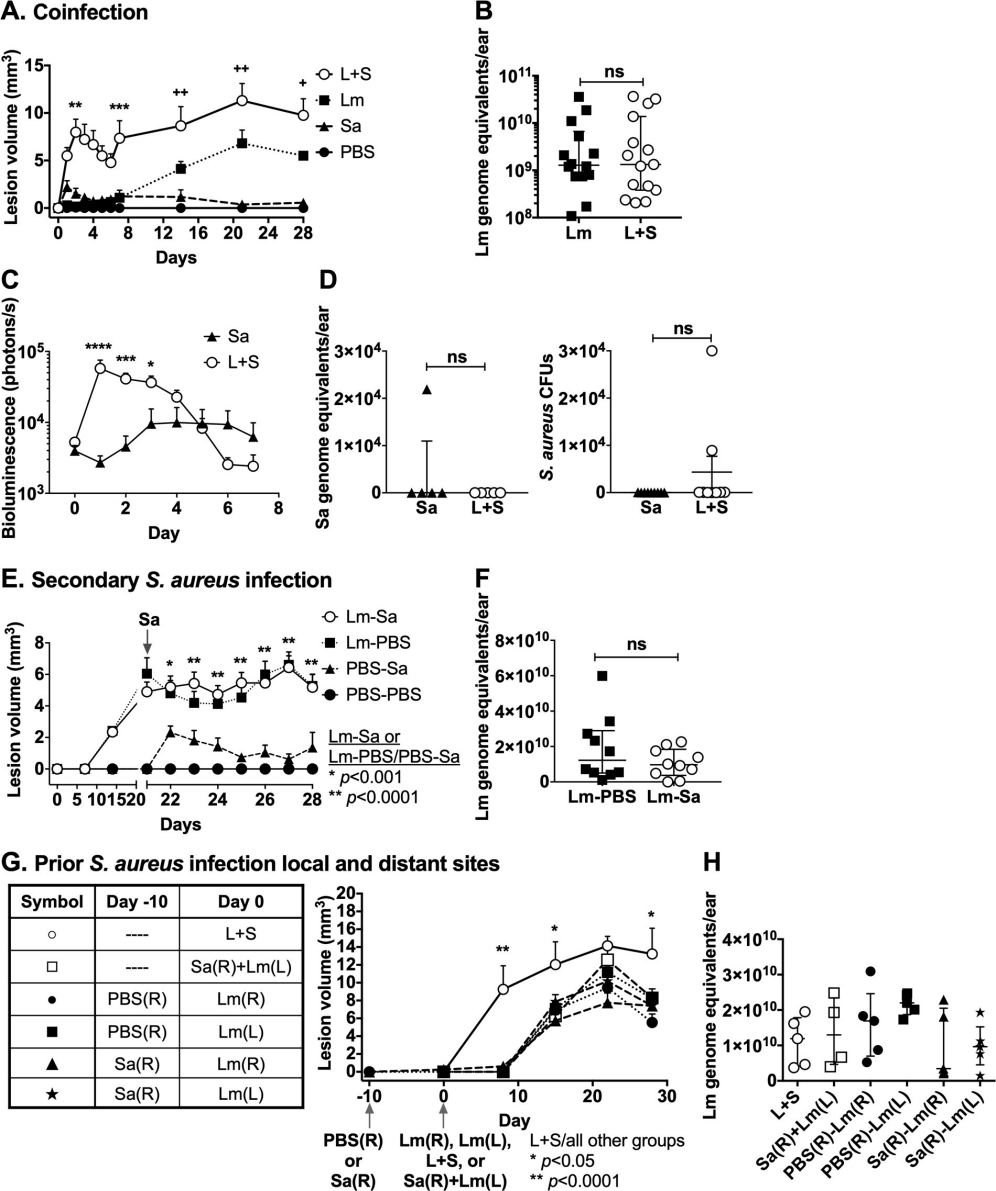
Sequential, prior, or simultaneous introduction of *S*. *aureus* during *L*. *major* infection. C57BL/6 mice were inoculated in ears with buffer (PBS), either WT Newman *S*. *aureus* or *S*. *aureus* LAC::*lux* (Sa), *L*. *major* (Lm) or *S*. *aureus* and *L*. *major* (L+S) with different timing or body sites of bacterial versus parasitic infection. Lesion sizes were measured throughout infection (panels A, E, G). Lm parasite loads were measured in the same mice at the end of experiments (panels B, F, H). (A) Simultaneous intradermal coinfection (L+S) in the C57BL/6 mouse ear pinna results in significantly greater lesion size than either organism alone. Data represent the mean ± SEM lesion volumes at different time points in three independent experiments, each with 4–5 mice/group. Asterisks (*) represent significance between Sa and L+S groups. Crosshairs (+) represent significance between Lm and L+S groups. (B) Parasite loads corresponding to single or coinfections in panel A. *S*. *aureus* infections were done using WT Newman strain *S*. *aureus*. Lm burdens were measured by qPCR of DNA from infected ears 28 days p.i. Data represent the mean ± SEM of three independent experiments, each with 4–5 mice/group. (C) *In vivo* imaging of Sa loads in single or coinfected mice: *S*. *aureus* LAC::*lux* was inoculated alone or with Lm. Bioluminescence corresponding to the load of live Sa was measured by *in vivo* imaging daily for 7 days. Data represent the mean ± SEM of two independent experiments, each with 5–10 mice/group. (D) (Left panel) Total Sa burden was measured by qPCR of DNA extracted from 28 days p.i. ears for one of the experiments in panel C. Data show the mean ± SD *S*. *aureus* genome equivalents in 5 mice/group. (Right panel) Live Sa burdens were also measured by counting colony-forming units (CFUs) after overnight growth from ear homogenates. Data represent the mean ± SEM CFU in the two independent experiments, each with 4–5 mice/group. (E) *L*. *major* infection prior to secondary *S*. *aureus* infection: mice were injected with Lm or phosphate buffered saline (PBS) intradermally in the ear on day 0. On day 21, the infection site was injected with either *S*. *aureus* to model superinfection of an established lesion (Lm-Sa), or PBS as a control (Lm-PBS). Data represent mean ± SEM lesion sizes at each time point from three independent experiments, each with 5 mice/group. Asterisks (*) indicate significance between PBS-Sa and Lm-Sa groups at all times beyond 21 days. (F) Parasite loads: Lm burdens 28 days p.i., corresponding to the Lm-PBS control or Lm-Sa sequential infection groups in panel A, were measured by qPCR in total ear DNA. Parasite burdens are expressed as Lm genome equivalents/ear. Data represent mean ± SEM from three independent experiments, each with 5 mice/group. (G) *S*. *aureus* infection prior to or simultaneous with *L*. *major* infection. (i) Prior: to model the importance of preexisting skin bacteria on lesion development, mice were injected with Sa or PBS intradermally in the right ear on day -10. On day 0, either the same ear or the opposite (left) ear was injected intradermally with Lm (closed symbols). (ii) Simultaneous: on day 0, groups of naïve mice were injected simultaneously with Lm and Sa (open symbols) in the same ear (L+S), or Sa in the right ear and Lm in the left ear (Sa(R)+Lm(L)). Lesion volume measurements are shown. Asterisks (*) represent significance between L+S coinfection group compared to all other groups. Data represented as the mean ± SD of one experiment with 5 mice/group. (H) *L*. *major* parasite burdens corresponding to panel C: despite significantly different lesion sizes, parasite loads were not significantly different between mice coinfected with L+S in the same or opposite ears, or by Sa prior to Lm infection. Data shown as the mean ± SD of one experiment with 5 mice/group. Unless otherwise indicated, *p < 0.05, **p < 0.01, ***p < 0.001, ****p < 0.0001, ns = not significant by one-way (H) or two-way (A, C, E, and G) ANOVA with Tukey’s post-test, or Student’s *t-*test (B, D, and F).

Bacteria can be introduced into the skin at the time of *Leishmania* inoculation by the bite of a sand fly or as CL lesions develop and ulcerate. This led us to use a murine model of CL to examine the phenotypic effects of bacteria introduced at different times or body site locations relative to the *L*. *major* parasitic infection. We chose to use *S*. *aureus*, a bacterium found in the sand fly gut [16] and commonly present at the site of human CL lesions [13–15], at a subclinical 10^4^ bacterial colony-forming units (CFUs) dose, which results in detectable but minimal swelling, and no ulceration in murine skin (S1 Fig). The simultaneous administration of *L*. *major* and *S*. *aureus* (L+S in figures) at a single site led to a significant and pronounced exacerbation of pathology compared to mice infected with either *L*. *major* or *S*. *aureus* alone (Lm or Sa in figures) (Fig 1). Administration of these organisms simultaneously at different body sites, sequentially at different body sites, or sequentially at the same body site in either order did not exacerbate pathologic changes compared to single infections. These results are specifically delineated in the Fig 1 legend.

Infections of C57BL/6 mice with *S*. *aureus* alone led to small lesions that peaked 2–3 days after infection and without ulceration. In contrast, *L*. *major* infections led to local swelling as the lesion developed gradually over 2–3 weeks and culminated in an ulcerated lesion by 3–4 weeks after infection (Fig 1A, 1E and 1G). Simultaneous inoculation of both *L*. *major* and *S*. *aureus* at the same site produced significantly exacerbated lesions, both during the first 7 days when *S*. *aureus* lesions formed and subsided, and throughout 2–4 weeks of coinfection when *L*. *major* lesions developed (Fig 1A). Parasite burdens after single or coinfection did not differ at days 3, 7 and 28 p.i., despite the widely divergent lesion sizes (Fig 1B, S2 Fig). A 10-fold lower dose (10^3^ CFUs) of *S*. *aureus* produced similarly exacerbated lesions during coinfection with *L*. *major*, and parasite burdens were also similar between these single and coinfection groups (S3 Fig). However, the early load of viable *S*. *aureus* was significantly higher in the presence of *L*. *major* and *S*. *aureus* coinfection compared to *S*. *aureus* alone, illustrated with light emissions from luminescent bacteria over the first 4 days of infection (Fig 1C). *S*. *aureus* loads, determined by qPCR and by CFU, were largely undetectable at day 28 when *L*. *major* lesions were large and often ulcerated (Fig 1D).

Other combinations of the timing or intradermal sites of microbial challenge did not lead to exacerbated lesion pathology or *L*. *major* parasite load (Fig 1E, 1F, 1G and 1H). As a model of late bacterial secondary infection, mice were intradermally infected with 10^6^ metacyclic *L*. *major* promastigotes. Ulcerating lesions formed over 21 days, after which late secondary infection was modeled by intralesional injection of 10^4^ CFUs of the MSSA *S*. *aureus* Newman strain. Secondary *S*. *aureus* infection did not alter the *L*. *major* lesion size throughout, or the parasite burden at 28 days post-*L*. *major* infection (Fig 1E and 1F).

Based on the hypothesis that systemic changes in dermal immunity resulted from prior cutaneous *S*. *aureus* infection, we tested the effect of augmenting bacterial burdens at different skin sites or prior to *L*. *major* challenge. *L*. *major* and *S*. *aureus* were inoculated simultaneously but in opposite ears, or *L*. *major* was inoculated into an ear that had been infected with *S*. *aureus* 10 days previously (Fig 1G and 1H). The data show that bacterial inoculation prior to or at a different site from that of *L*. *major* inoculation had no effect on the size of parasite-induced skin lesions. Consistently, *L*. *major* burdens were not significantly different among any of the infection groups at 28 days post-*L*. *major* infection (Fig 1H).

### *L*. *major* coinfection with *S*. *aureus* exacerbates lesion development and increases the presence of tissue neutrophils

**Fig 2:**
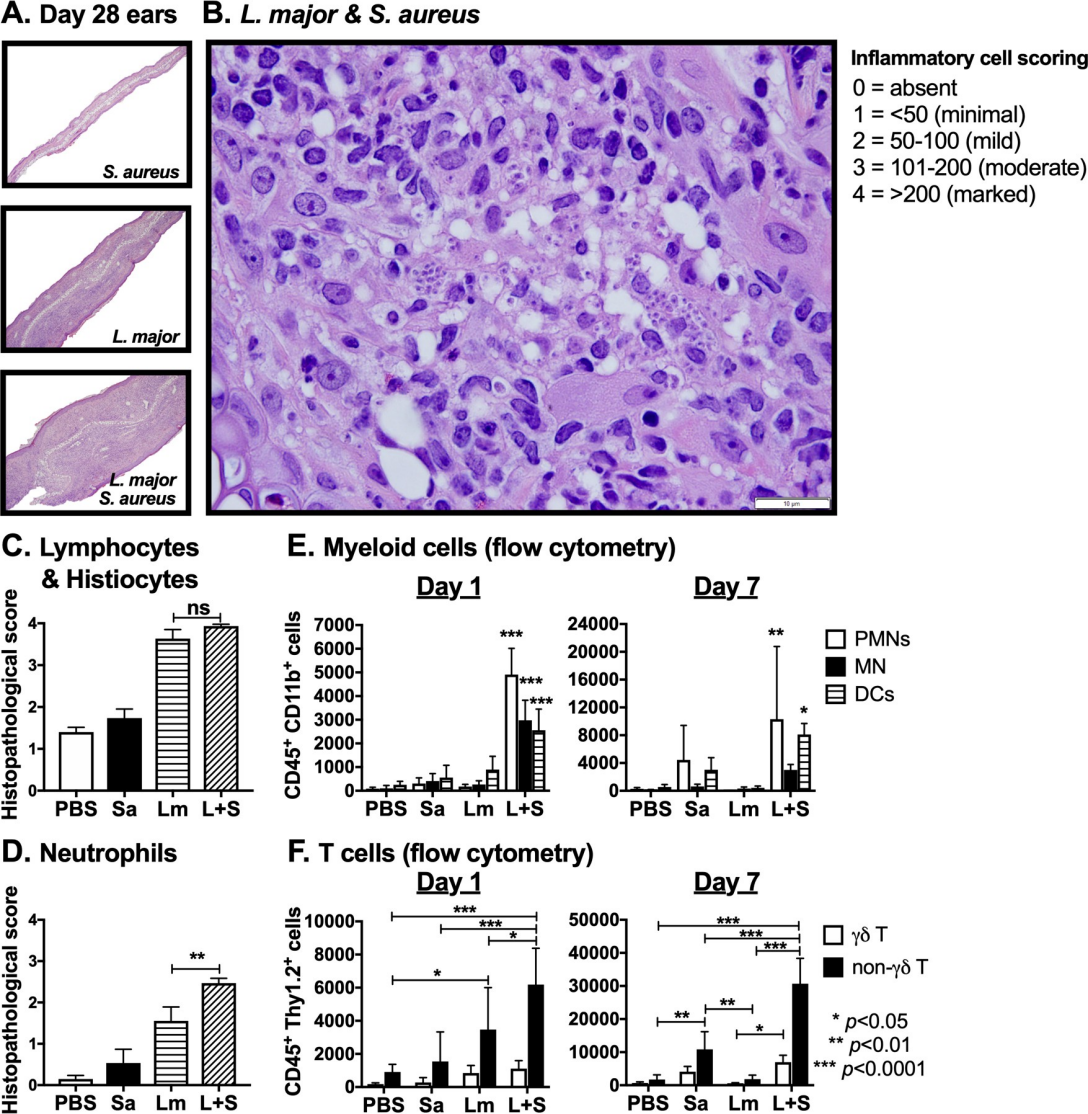
*L*. *major-S*. *aureus* coinfection results in greater neutrophil infiltration than *L*. *major* alone. (A) Low-powered image (100x) of paraffin-embedded hematoxylin and eosin-stained sections of ears from mice intradermally injected with *S*. *aureus* (Sa), *L*. *major* (Lm), or co-inoculated with both (L+S) 28 days p.i. (B) High-powered image (1000x) of histological section from coinfected ear. White bar represents 10 μm. (C) Histological inflammatory cell scores for lymphocytes and histiocytes or (D) neutrophils from ears 28 days p.i. were calculated as the average score of four high-powered (400x) fields per ear. (E) Numbers of neutrophils (PMNs) (CD45^+^ CD11b^+^ Ly6G^hi^ Ly6C^int^), inflammatory monocytes (MN) (CD45^+^ CD11b^+^ Ly6G^-^ Ly6C^hi^), and myeloid dendritic cells (DC) (CD45^+^ CD11b^+^ CD11c^+^), shown as absolute cell numbers in samples derived from inoculated ears at days 1 and 7 p.i. was determined by flow cytometry. (F) Numbers of γδ (CD45^+^ Thy1.2^+^ γδ TCR^+^) and non-γδ (CD45^+^ Thy1.2^+^ γδ TCR^-^) T cell numbers from the ears at days 1 and 7 p.i. was determined by analyzing single cells suspensions from whole ear samples by flow cytometry. Data are shown as the mean ± SEM of three separate experiments, each with 4–5 mice/group (A, B, C, and D), or as the mean ± SD of 5 mice/group in one of three representative experiments (E and F). *p < 0.05, **p < 0.01, ***p < 0.0001, ns = not significant by one-way ANOVA (C and D) or two-way ANOVA (E and F) with Tukey post-test for multiple comparisons.

Since neither pathogen burden differed between infection groups at 28 days post-coinfection, we hypothesized that changes in immune cell infiltration into coinfected ears exacerbated *L*. *major* lesions. Lesion pathology at low magnification revealed that ears that had been coinfected demonstrated increased ear thickness and greater inflammatory cell infiltrates than did tissue histology in all other infection groups (Fig 2A). Semi-quantitative histologic scores showed that lymphocytes and histiocytes predominated in both *L*. *major* singly infected and *L*. *major-S*. *aureus* coinfected ears at day 28 p.i. (Fig 2B and 2C). Unexpectedly in light of the chronicity of infection (day 28), there were significantly more neutrophils at day 28 p.i. in *L*. *major-S*. *aureus* coinfected ears than in single *L*. *major* infections, which correlated with enhanced lesion size (Fig 2D).

To confirm the identity of inflammatory cells in ears of coinfected or singly infected mice, we stained cells recovered from lesions for inflammatory cell surface markers at different times of infection (Fig 2E and 2F). Significantly more neutrophils (CD45^+^ CD11b^+^ Ly6G^hi^ Ly6C^int^), inflammatory monocytes (CD45^+^ CD11b^+^ Ly6G^-^ Ly6C^hi^), myeloid dendritic cells (CD45^+^ CD11b^+^ CD11c^+^), and γδ T cells (CD45^+^ Thy1.2^+^ γδ TCR^+^) were recovered from coinfected ears at day 1 p.i. compared to PBS, *S*. *aureus*, or *L*. *major* singly infected, or PBS inoculated mice (Fig 2E and 2F). There were also more neutrophils, myeloid dendritic cells, γδ and non-γδ T cells in coinfected ears on day 7 p.i., compared to PBS or *L*. *major* infection groups (Fig 2E and 2F).

### Neutrophils recovered from *L*. *major-S*. *aureus* coinfected lesions exhibit similar phagocytosis of microbes and NADPH oxidase activity, but are mostly apoptotic and present in an environment that downregulates efferocytosis

**Fig 3:**
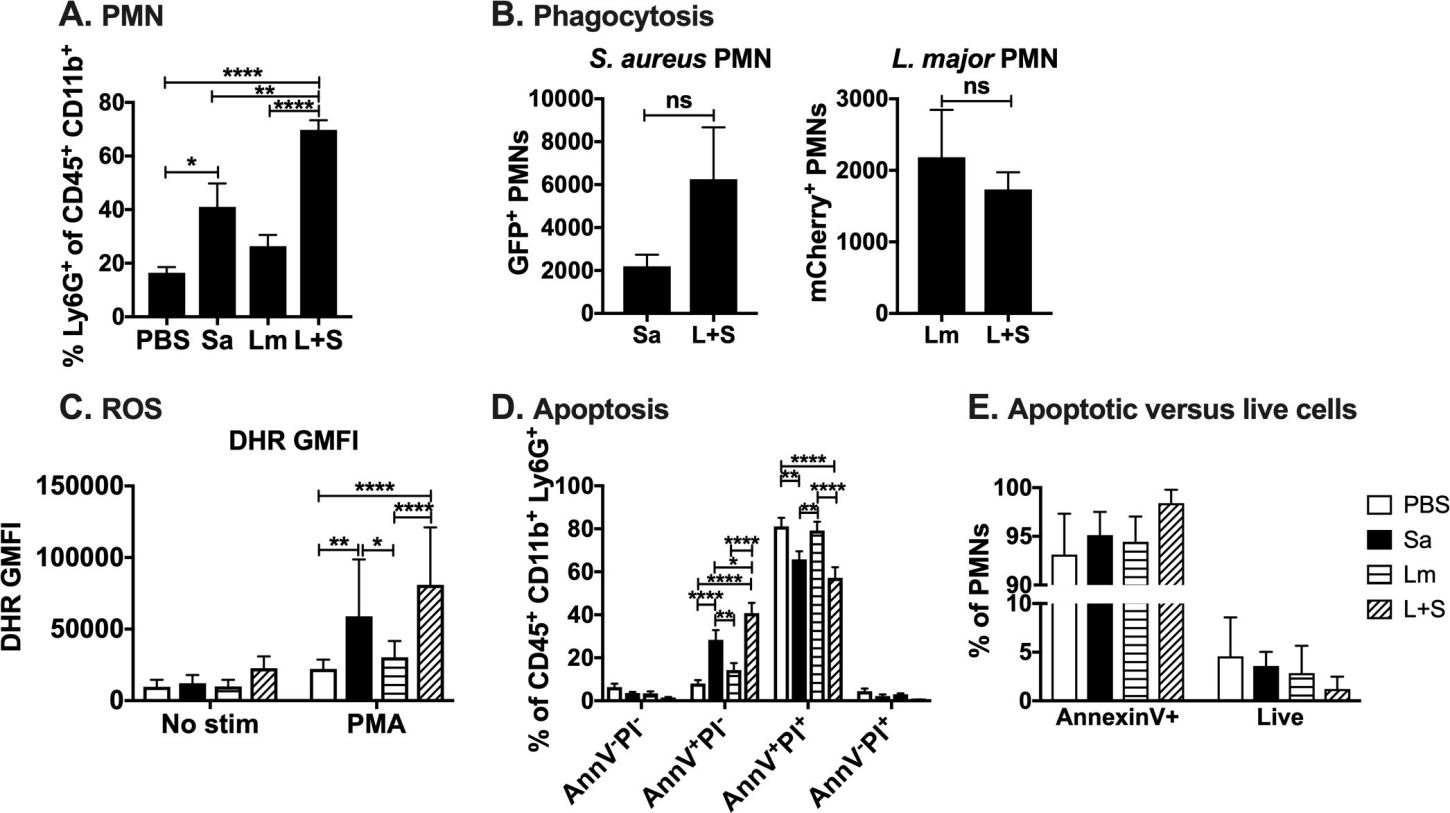
Neutrophils in *L*. *major-S*. *aureus* coinfected lesions can phagocytose pathogens and have functional NADPH oxidase but are mostly apoptotic. (A) Percentage of myeloid cells that are neutrophils from mice injected intradermally in the ear with phosphate buffered saline (PBS), *L*. *major* IA-2 (Lm), *S*. *aureus* Newman (Sa), or coinfected with *L*. *major* and *S*. *aureus* (L+S) for one day. (B) Numbers of polymorphonuclear cells (PMNs) positive for *S*. *aureus* LAC GFP or *L*. *major* IA-2 luc mCherry in singly infected versus coinfected lesions. (C) Dihydrorhodamine (DHR) geometric mean fluorescence intensity (GMFI) of neutrophils stimulated with PMA *ex vivo* in the presence of DHR to assess NADPH oxidase activity. (D) Annexin V (AnnV) and propidium iodide (PI) staining of neutrophils from PBS, *S*. *aureus*, *L*. *major*, or coinfected lesions. (E) Percentage of apoptotic (Annexin V^+^) versus live (AnnV^-^PI^-^) neutrophils from day 1 p.i. PBS, *S*. *aureus*, *L*. *major*, or coinfected ears. Data are represented as the mean ± SEM of two replicate experiments each with 5 mice/group. *p < 0.05, **p < 0.01, ***p < 0.001, ****p < 0.0001, ns = not significant by Student’s *t* test (B), or one-way (A, C) or two-way ANOVA (D) with Tukey’s post-test.

Early *L*. *major-S*. *aureus* coinfected lesions exhibited enhanced *S*. *aureus* replication despite the presence of more neutrophils (Fig 1C, Fig 2E). Because neutrophils are critical for the clearance of cutaneous *S*. *aureus* infections and wound resolution [38–40] it seemed paradoxical that there were more neutrophils yet a greater burden of *S*. *aureus* during the first three days of coinfection with *L*. *major* (Fig 1C, Figs 2E and 3A). To understand the mechanisms underlying this apparent paradox, we first explored the hypothesis that neutrophil phagocytic or microbicidal capacities were defective during coinfection. To assess the phagocytic capacity of recruited neutrophils, we used *S*. *aureus* LAC expressing green fluorescent protein (GFP) and *L*. *major* IA-2 expressing luciferase and mCherry to detect selectively each microbe after intradermal injection into mouse ears. Neutrophils from coinfected lesions one day p.i. phagocytosed either pathogen (Fig 3B). The phagocytosis of *S*. *aureus* or *L*. *major* was the same in the absence or presence of coinfection. Thus, coinfection did not compromise the capacity of neutrophils to ingest the pathogens.

Because neutrophil killing of *S*. *aureus* predominantly depends on a functional phagocyte NADPH oxidase, we used flow cytometric analysis of dihydrorhodamine (DHR) oxidation to assess the ability of recruited neutrophils to generate reactive oxygen species in response to the soluble agonist, phorbol myristate acetate (PMA) [41]. Based on the average DHR GMFI of PMA-stimulated neutrophils from the infection site, neutrophils containing either microbe had functional NADPH oxidases, with activities highest in neutrophils recovered from lesions containing *S*. *aureus* (Fig 3C). Taken together, these data suggest that the presence of more neutrophils in the lesions from coinfected tissue did not reflect defective antimicrobial capacity of the recruited neutrophils.

We next tested the hypothesis that the excess of neutrophils in coinfected lesions might reflect a defect in the resolution of the inflammatory response. That is, a failure of the recruited neutrophils to undergo apoptosis and be efferocytosed by tissue macrophages. To test this hypothesis, we quantified apoptotic neutrophils by annexin V (AnnV) staining and measured the expression of efferocytosis-related genes by RT-qPCR.

AnnV and propidium iodide (PI) staining of ear cells from all groups of mice coinfected one day p.i. revealed a greater percentage of neutrophils undergoing apoptosis (AnnV^+^PI^-^) or necrotic cells (AnnV^+^PI^+^), compared to cells from mice injected with PBS, *L*. *major*, or *S*. *aureus* alone (Fig 3D and 3E, S4 Fig). Given that coinfected ears had over 9-fold greater absolute numbers of neutrophils than ears infected with either pathogen alone (Fig 2E), this AnnV staining indicates the majority of those neutrophils are apoptotic (Fig 3D and 3E). Thus, significantly more neutrophils recovered from coinfected lesions were apoptotic compared to all other infection groups.

Resolution of inflammation depends in part on efferocytosis, a process that limits inflammatory and promotes anti-inflammatory responses through the uptake of apoptotic cells by tissue macrophages [42, 43]. Many ligand-receptor pairs initiate uptake of apoptotic cells, but a common ligand is phosphatidylserine exposed on the apoptotic or “to-be-eaten” cell. The potential biological importance of greater numbers of cutaneous AnnV^+^ neutrophils led us to examine the local expression of genes often associated with efferocytosis, reasoning that the transcripts will parallel the immune “tone” or abundant immune functions active locally. Total RNA content in ears of *S*. *aureus* and/or *L*. *major* singly- or coinfected ears or uninfected control ears were extracted 3 days p.i. RT-qPCR assays revealed lower total mRNA present in *S*. *aureus* singly and coinfected ears compared to *L*. *major-*infected ears for genes such as immunoregulatory cytokines IL-10 and IL-13, and matrix metalloproteinase-9 (MMP9), an enzyme important for cutaneous wound healing (S5A & S5B Fig). Additionally, *S*. *aureus-*infected ears had lower mRNA levels for LXRα and PPARδ, two nuclear receptors associated with efferocytosis (S5C Fig). Taken together, these data suggest that many of the neutrophils recovered from skin coinfected with *S*. *aureus* were apoptotic. In a setting where factors important for efferocytosis and resolution of inflammation were downregulated, the recruited neutrophils would accumulate but be ineffective at clearing infection, all of which may promote *S*. *aureus* survival and replication. In fact, *in vitro* studies demonstrate that human neutrophils harboring viable *S*. *aureus* display increased AnnV binding but are not efferocytosed by human monocyte-derived macrophages [44].

### T cells from *L*. *major-S*. *aureus* coinfected animals produce *L*. *major* antigen-induced IL-17A

The presence of more immune cells in the early stages, and the persistent neutrophil infiltrate in lesions at the late stages of coinfection led us to examine potential differences in cytokines expressed. After 4 weeks of infection, most transcripts encoding inflammatory or modulatory cytokines were similar in abundance in ear tissues extracted from *L*. *major*-infected and coinfected groups. These included transcripts encoding innate, Type 1, and Type 2 cytokines or chemokines (S6 Fig and S1 Table).

**Fig 4:**
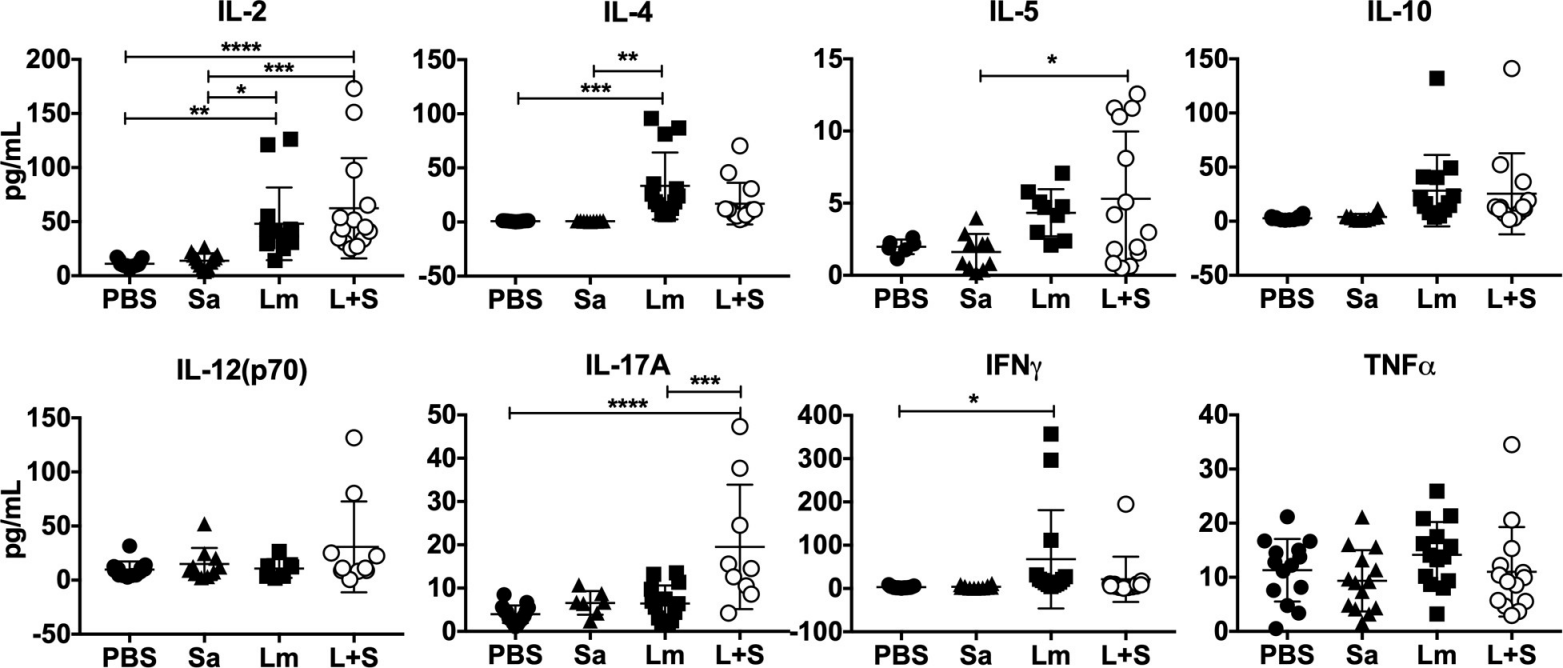
Immune cells from *L*. *major*-*S*. *aureus* coinfected mice produce similar levels of Th1- and Th2-type cytokines, but more IL-17A than singly infected mice in response to *L*. *major* antigen. Draining lymph nodes from mice day 28 p.i. were re-stimulated with live *L*. *major* promastigote antigen for 72 hours and cytokine levels in culture supernatants were assayed by multiplex ELISA. Data represent the mean ± SEM of three replicate experiments, each with 4–5 mice/group. *p < 0.05, **p < 0.01, ***p < 0.001, ****p < 0.0001, ns = not significant by one-way ANOVA with Tukey’s post-test. PBS = phosphate buffered saline, Sa = *S*. *aureus*, Lm = *L*. *major*, L+S = *L*. *major + S*. *aureus*.

To examine antigen-responsive adaptive immune responses, we restimulated draining lymph node (LN) cells from infected mice with total *Leishmania* antigen and measured cytokines released in a fluorescent bead-based multiplex cytokine assay. Although *L*. *major* infection led to increased IFNγ and IL-4 compared to uninfected, in PBS-injected mice, the only Type 1 (Th1-type) or Type 2 (Th2-type) cytokines elevated above single infections were IL-2 and IL-5 (Fig 4). These results fail to implicate Type 1 and Type 2 T cells in the exacerbated pathology of coinfection. There were also no significant differences in IL-10, IL-12(p70), or TNFα released by cells from any of the infection groups. However, IL-17A was found at significantly higher concentrations in supernatants of cells from antigen-stimulated coinfected draining lymph nodes compared to *L*. *major*-infected alone draining lymph nodes (Fig 4). This result raises the possibility that *Leishmania-*specific Type 17 helper T cells (Th17) develop during coinfection but not during single infections in the C57BL/6 model.

### γδ T cells and Th17 cells produce IL-17A with different kinetics during *L*. *major-S*. *aureus* coinfection

**Fig 5:**
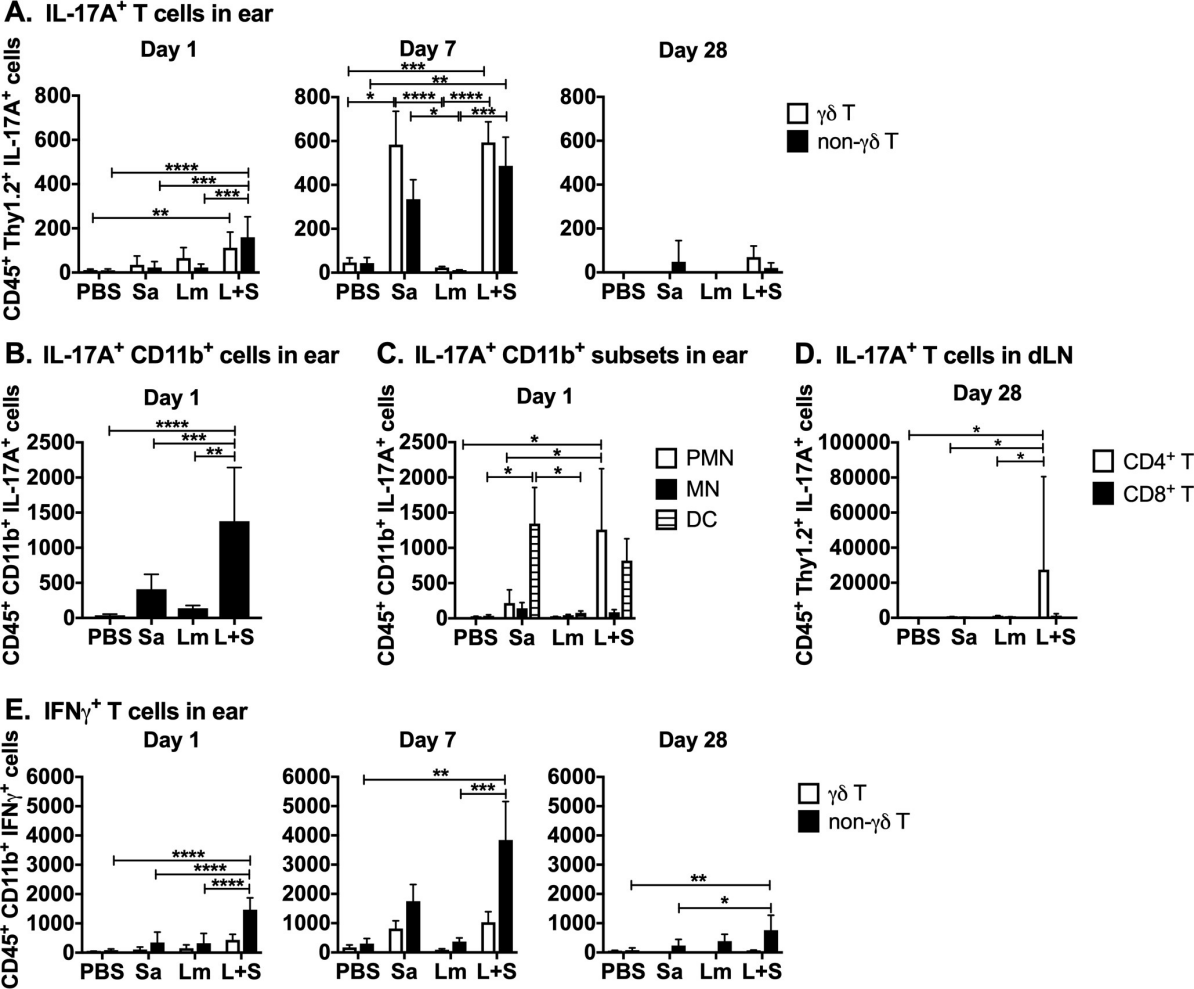
*L*. *major* and *S*. *aureus* coinfection results in elevated levels of immune cells producing proinflammatory cytokines. Ears and draining lymph nodes of mice were homogenized, cultured for 4–5 hours with BFA, PMA, and ionomycin, and stained for surface markers, intracellular IL-17A or IFNγ, and analyzed by flow cytometry. Stained cells were gated on surface markers followed by intracellular cytokines. (A) IL-17A^+^ γδ (CD45^+^ Thy1.2^+^ γδ TCR^+^) and non-γδ (CD45^+^ Thy1.2^+^ γδ TCR^-^) T cell numbers from the ears at days 1, 7, and 28 p.i. (B) Numbers of IL-17A^+^ cells co-staining with CD11b from ears 1 day p.i. (C) Numbers of IL-17A^+^ cells co-staining CD45^+^ CD11b^+^ Ly6G^hi^ Ly6C^int^ (PMN), CD45^+^ CD11b^+^ Ly6G^-^ Ly6C^hi^ (MN), or CD45^+^ CD11b^+^ CD11c^+^ (DC) from the ears at 1 day p.i. (D) Numbers of IL-17A^+^ CD4 (CD11b^-^ Thy1.2^+^ CD4^+^ CD8^-^) and CD8 (CD11b^-^ Thy1.2^+^ CD4^-^ CD8^+^) T cells from draining lymph nodes at day 28 p.i. (E) Numbers of IFNγ^+^ γδ (CD11b^-^ Thy1.2^+^ γδ TCR^+^) and non-γδ (CD11b^-^ Thy1.2^+^ γδ TCR^-^) T cells from the ears at days 1, 7, and 28 p.i. Data at day 7 and CD11b^+^ cell data (panels A, B, C, & E) are shown as the mean ± SEM of two pooled experiments, each with 3–6 mice/group. T cell data at day 1 and 28 (panels A, D, & E) are representative of one experiment and shown as the mean ± SD of 5 mice/group. *p < 0.05, **p < 0.01, ***p < 0.001, ****p < 0.0001 by one-way ANOVA (B) or two-way ANOVA (A, C, D, and E) with Tukey’s post-test. PBS = phosphate buffered saline, Sa = *S*. *aureus*, Lm = *L*. *major*, L+S = *L*. *major + S*. *aureus*.

In order to identify IL-17A producing cells during *L*. *major-S*. *aureus* coinfection, we performed intracellular cytokine and surface marker staining on LN or ear-derived inflammatory cells extracted one day p.i. Inflammatory cells were incubated in PMA and ionomycin in the presence of Brefeldin A, and then stained and analyzed by flow cytometry using the gating strategy shown in S7, S8, and S9 Figs. Intracellular and surface staining revealed greater numbers of IL-17A-producing γδ T cells and non-γδ T cells observed in coinfected ears at both 1 and 7 days p.i. (Fig 5A). IL-17A^+^ CD11b^+^ cells were also detected in coinfected ears at 1 day p.i. (Fig 5B and 5C). We also observed more IFNγ-producing non-γδ T cells in coinfected compared to singly-infected ears at days 1 and 7 p.i. (Fig 5E), which may implicate innate lymphoid cells at the infection site early during coinfection. After 28 days of infection, there were few IL-17A^+^ cells in the ears of all infection groups (Fig 5A). However, there were significantly more IL-17A^+^ CD4^+^ (non-γδ) T cells in the draining LNs of coinfected mice compared to mice infected with either *L*. *major* or *S*. *aureus* alone at 28 days p.i. (Fig 5D). These data suggest the source of IL-17A differs in the acute versus chronic *L*. *major-S*. *aureus* coinfected lesions.

### *L*. *major-S*. *aureus* coinfection promotes pro-IL-1β expression

**Fig 6:**
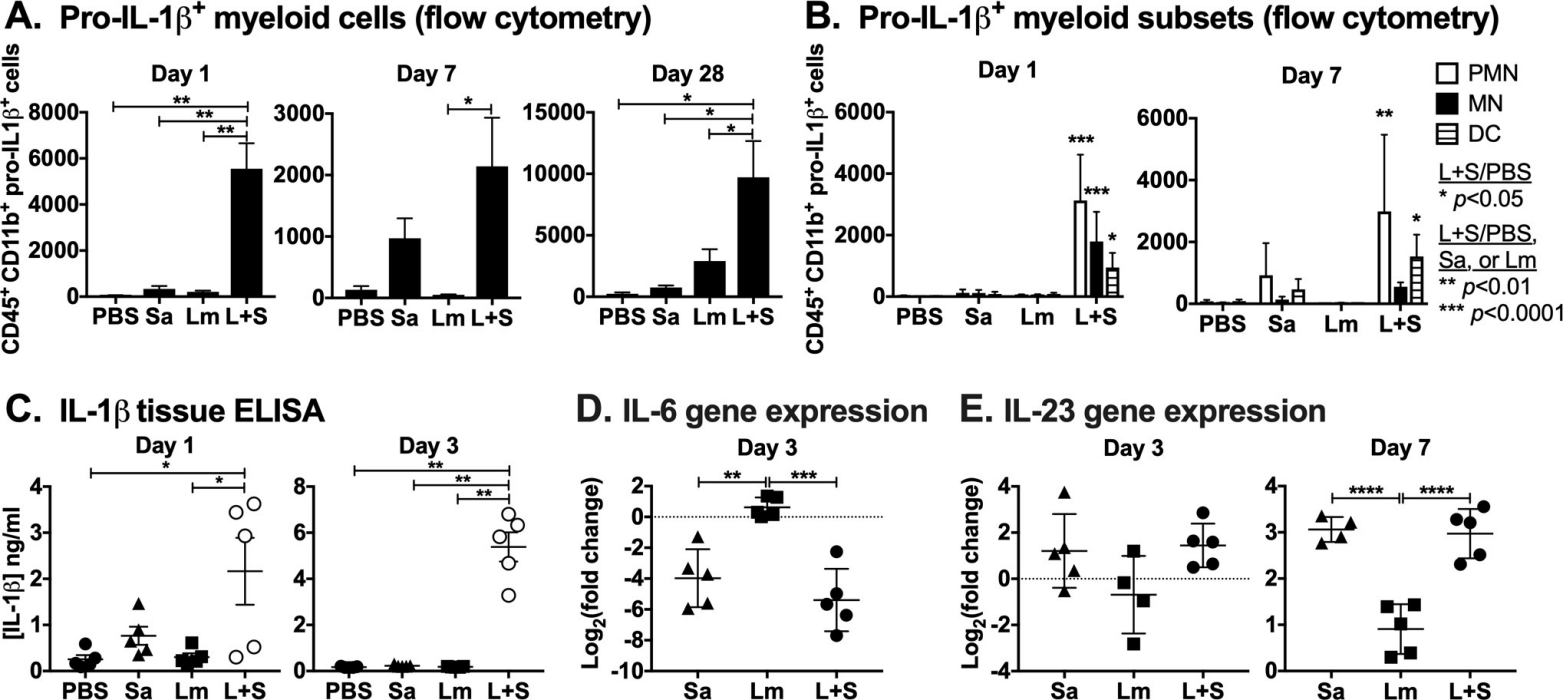
*L*. *major-S*. *aureus* coinfection results in increased pro-IL-1β expressing myeloid cells, higher IL-1β levels, and greater IL-23 gene expression in infected tissues. Infected mice ears were homogenized and (A) cultured for 4–5 hours with BFA, PMA, and ionomycin, stained for surface markers and intracellular pro-IL1β, and analyzed by flow cytometry for (B) numbers of pro-IL-1β^+^ cells co-staining CD45^+^ CD11b^+^ Ly6G^hi^ Ly6C^int^ (PMN), CD45^+^ CD11b^+^ Ly6G^-^ Ly6C^hi^ cells (MN), or CD45^+^ CD11b^+^ CD11c^+^ (DC) from the ears at days 1 and 7 p.i. (C) Supernatants from homogenized ear tissues were analyzed by ELISA for IL-1β concentration. (D) IL-6 gene expression from ears 3 days p.i. (E) IL-23 gene expression from ears 3 and 7 days p.i. Data from days 1 and 3 (A-C) and panels D & E represent one experiment and are shown as the mean ± SD of 5 mice/group. Data from day 7 (A & B) from two replicate experiments are shown as the mean ± SEM of 3–6 mice/group. *p < 0.05, **p < 0.0001 by one-way ANOVA with Tukey’s post-test. PBS = phosphate buffered saline, Sa = *S*. *aureus*, Lm = *L*. *major*, L+S = *L*. *major + S*. *aureus*.

The correlation between *L*. *major-S*. *aureus* coinfection exacerbation and elevated IL-17A from γδ T cells and Th17 cells led us to examine potential upstream factors promoting an IL-17A response in different cell types. Inflammasome-derived IL-1β can promote the expression of IL-17A by γδ T cells [45, 46] and Th17 cells [47, 48], as well as the differentiation of naïve T cells into Th17 cells [49]. Pro-IL-1β is upregulated at a transcriptional level by priming conditions, and secreted IL-1β must undergo proteolytic cleavage by caspase-1 in order to be biologically active [50]. Additionally, IL-6 and particularly IL-23 are important for the differentiation and maintenance of Th17 cells [51, 52]. We used flow cytometry to measure pro-IL-1β abundance in inflammatory cells extracted from the ears of singly or coinfected mice. After 1 or 7 days p.i., we observed significantly more CD45^+^ CD11b^+^ myeloid cells with intracellular pro-IL-1β in coinfected ears compared to *S*. *aureus*-infected ears after 1 or 28 days, and compared to *L*. *major*-infected ears after 1, 7, or 28 days p.i. (Fig 6A). Most of the pro-IL-1β^+^ CD11b^+^ cells during early infection showed surface marker staining consistent with neutrophils (CD45^+^ CD11b^+^ Ly6G^hi^ Ly6C^int^) (Fig 6B). The abundance of active IL-1β by ELISA in coinfected ears at day 1 and day 3 p.i. compared to all other infection groups (Fig 6C). These increased levels of IL-1β early during coinfection may be due to the elevated *S*. *aureus* burdens observed during the early stages of coinfection with *L*. *major*. Expression of IL-6 at day 3, and IL-23 at days 3 and 7 was assessed by RT-qPCR. These assays revealed significantly greater abundance of IL-6 mRNA in the ears of the *L*. *major* single infection group, but lower IL-23 mRNA in *L*. *major* single infected mice, compared to myeloid cells from the ears of mice infected either with *S*. *aureus* alone or coinfected with *L*. *major* and *S*. *aureus* (Fig 6D and 6E). These observations suggest that IL-23 and IL-1β responding to *S*. *aureus* in the skin might contribute to enhanced production of IL-17A from innate T cells at early times of infection. As a corollary, augmented IL-17A early during infection might drive the differentiation of naïve T cells into Th17 cells in the later stages of coinfection.

### Phenotype of *L*. *major-S*. *aureus* coinfection is not *S*. *aureus* strain-specific

**Fig 7:**
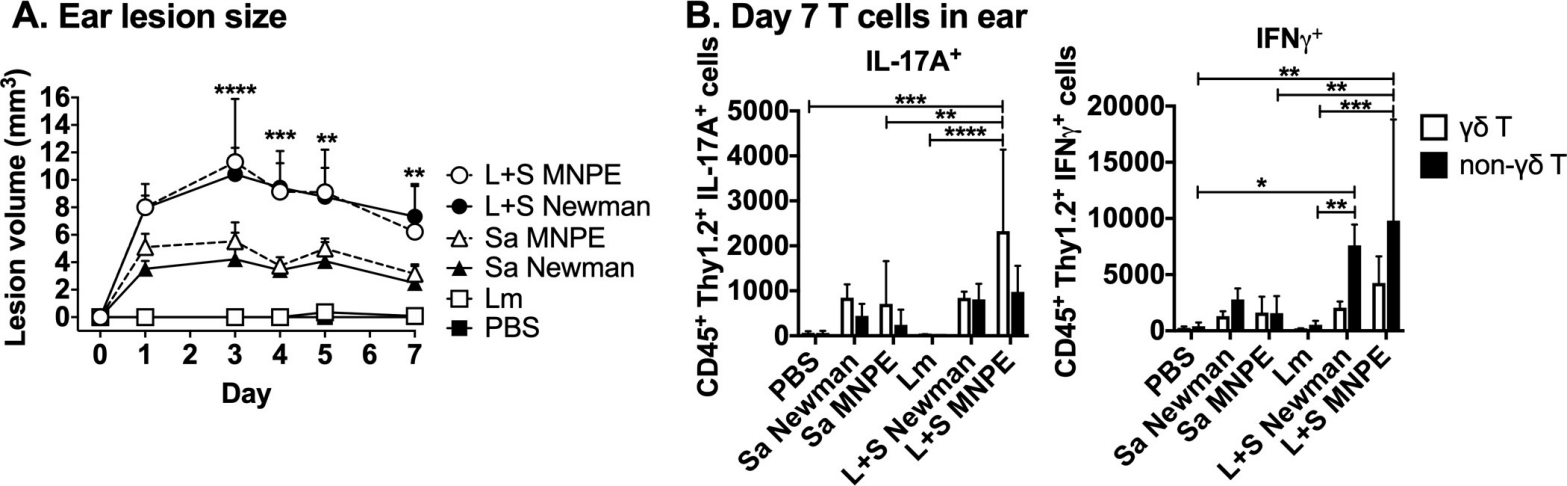
Coinfection with *S*. *aureus* MNPE, a USA200 TSST^+^ clinical strain, recapitulates early *L*. *major-S*. *aureus* Newman lesion exacerbation and cytokine production. Mice were injected intradermally in the ear with phosphate buffered saline (PBS), *L*. *major* (Lm), *S*. *aureus* Newman (Sa Newman), *S*. *aureus* MNPE (Sa MNPE) or co-inoculated with *L*. *major* and either *S*. *aureus* strain (L+S Newman or L+S MNPE) and ear lesion volume was measured for 7 days. On day 7 p.i., single cell suspensions from the ears were obtained, treated with BFA, PMA, and ionomycin for 4–5 hours, stained for surface markers and intracellular cytokines, and analyzed by flow cytometry. (A) Lesion volume of ears coinfected with *L*. *major* and *S*. *aureus* MNPE compared to those coinfected with *S*. *aureus* Newman MSSA. (B) IL-17A^+^ or IFNγ^+^ γδ (CD45^+^ Thy1.2^+^ γδ TCR^+^) and non-γδ (CD45^+^ Thy1.2^+^ γδ TCR^-^) T cell numbers in ears. Data represent the mean ± SD of one experiment with 5–6 mice/group. Lesion size data are representative of two independent experiments. *p < 0.05, **p < 0.01, ***p < 0.001, ****p < 0.0001 by one-way ANOVA with Tukey’s post-test.

The coinfection phenotype was not limited to the *S*. *aureus* Newman strain. *L*. *major* IA-2 coinfections with *S*. *aureus* MNPE, a methicillin-sensitive USA200 strain that produces the superantigen toxic shock syndrome toxin (TSST-1), recapitulated the lesion exacerbation seen with *S*. *aureus* Newman MSSA (Fig 7A). These lesions revealed more highly elevated IL-17A production by γδ T cells in coinfection (Fig 7B). Thus, two clinically relevant *S*. *aureus* isolates that express different secreted toxins exacerbate lesions in a murine model of cutaneous leishmaniasis in a similar manner.

### Neutralization of IL-17A partially ameliorates early lesion exacerbation whereas IL-1β neutralization further exacerbates coinfected lesions

**Fig 8:**
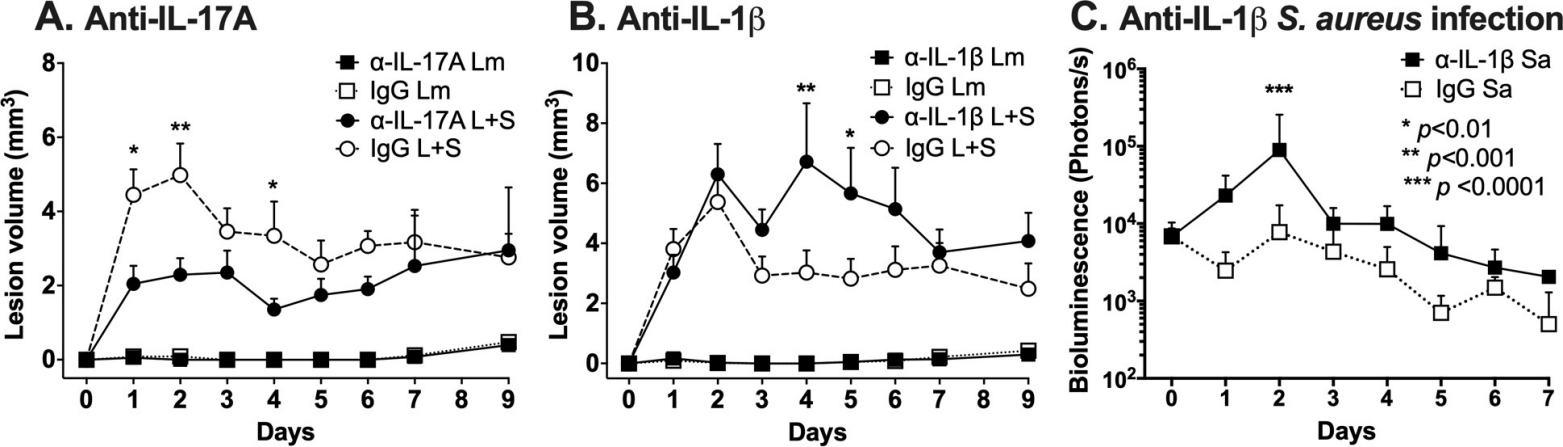
Anti-IL-17A neutralizing antibodies partially ameliorate early coinfection lesion exacerbation while anti-IL-1β antibodies further exacerbate lesions. Mice were injected intraperitoneally with 0.5 mg neutralizing antibody or isotype control antibody one day prior to intradermal ear injection with *L*. *major* (Lm) or *L*. *major* and *S*. *aureus* (L+S). Neutralizing antibody was administered one day prior to infection and every three days for 9 days. Ear lesion volumes during coinfection in mice treated with (A) anti-IL-17A or MOPC-21 isotype antibodies, or (B) anti-IL-1β or polyclonal Armenian hamster IgG isotype antibodies are shown. Data represent the mean ± SEM of two independent experiments, each with 5 mice per group. IgG L+S versus anti-IL-17A L+S or anti-IL-1β L+S comparisons: *p < 0.01, **p < 0.001, ***p < 0.0001 by two-way ANOVA with Tukey’s post-test. (C) Bacterial load by *in vivo* bioluminescence imaging of mice treated with anti-IL-1β or polyclonal Armenian hamster IgG isotype antibodies and infected with 10^4^ CFU *S*. *aureus* LAC::*lux* alone. Data represent the mean ± SD of one experiment with 5 mice per group. ***p < 0.0001 by two-way ANOVA with Tukey’s post-test.

We hypothesized that the increased production of IL-17A during *L*. *major-S*. *aureus* coinfection may contribute to the observed enhanced pathology, and that IL-17A expression might be stimulated by IL-1β. We tested this hypothesis by treating *L*. *major* singly infected or *L*. *major-S*. *aureus* coinfected mice with neutralizing anti-IL-17A or anti-IL-1β antibodies through the first two weeks infection (Fig 8A and 8B). Coinfected groups treated with anti-IL-17A antibody developed two-fold reduction in early lesion volumes compared to mice treated with isotype control antibody at day 2 p.i. (Fig 8A), consistent with a lesion-exacerbating effect of IL-17A. However, coinfected groups treated with anti-IL-1β antibody developed two-fold larger lesion volumes compared to isotype control treated mice at day 4 p.i. (Fig 8B). This was not due to an inability of anti-IL-1β antibodies to reach the ear skin site, although antibodies were only able to decrease the level of IL-1β in the ears by 64% (S10 Fig). Neutralizing antibodies to IL-17A or IL-1β had no effect on ear lesions of mice infected with *L*. *major* alone, suggesting they influence immune responses elicited in the presence of bacteria. The anti-IL-17A data suggest that IL-17A is partially responsible for the lesion exacerbation that occurs during coinfection, but this does not exclude a contribution of other immune mediators and bacterial or parasitic factors to the development of disease.

We hypothesized that the results of neutralizing IL-1β may reflect competing actions of IL-1β in this model. IL-1β promotes clearance of *S*. *aureus* from skin lesions, an observation illustrated by our control mice singly infected with *S*. *aureus* alone, in which anti-IL-1β caused a significant exacerbation of bacterial loads (Fig 8C). A second effect of IL-1β may be to induce IL-17A, which in turn would recruit neutrophils to lesion sites. We surmise that at the early time points shown in our study, the defective bacterial killing in the face of diminished IL-1β in the vicinity might dominate over the effects of lowering IL-17A responses in terms of lesion size. The data suggest that the several proinflammatory functions of IL-1β may participate in controlling bacterial replication and lesion size during the early phases of cutaneous *L*. *major-S*. *aureus* coinfection.

## Discussion

Determinants of the outcome of host defense against infection include input from both host and microbe and interactions that are bidirectional. The immune environment into which a pathogen is introduced plays a critical role in the fate of microbial infection [4]. Recent studies on microbiomes have demonstrated that commensal microbes present at a non-sterile body site, such as the skin, modulate local immune environments [4–6, 15, 53]. Adding to these complex and reciprocal interactions, studies on polymicrobial infections have revealed a pattern of synergy between coinfecting organisms with overlapping biogeography [54–57]. In the case of vector-borne infectious diseases such as cutaneous leishmaniasis, microbiota from the non-sterile gut environment of the insect vector can also be introduced in concert with the parasite into mammalian skin [12, 16]. The current study tested the hypothesis that the simultaneous presence of bacteria on mammalian skin at the site of inoculation with *Leishmania* spp. modifies the local immune response, either enhancing parasite killing or exacerbating leishmanial disease.

Simultaneous intradermal coinfection of C57BL/6 mice with *L*. *major* and *S*. *aureus* resulted in exacerbation of lesions characterized by the presence of more neutrophils. The timing and site of coinfection were critical in our studies, because coincident infection with *S*. *aureus* inoculated prior to *L*. *major*, or concurrently at a different body site, did not alter the course of severity of *L*. *major* lesion development or burdens. These findings mirror results of a study of concurrent infections of hamsters with *Leishmania braziliensis panamensis* and either *S*. *aureus* or *Pasteurella* multocida, which demonstrated enhanced early lesion size and bacterial burdens, although late lesions were not changed [58]. Other models of cutaneous leishmaniasis, including studies of gene knockout mice (NLRP10 and TNFRp55) [59–61] describe enhanced lesion severity without an associated increase in *L*. *major* burden [59, 62].

In contrast to the absence of an increase in parasite load, augmented lesion size during early coinfection correlated with elevated *S*. *aureus* burdens in the first three days of coinfection, despite the enhanced numbers of neutrophils, cells that typically ingest, kill, and eliminate *S*. *aureus* from tissues. Compared to single infections, *L*. *major-S*. *aureus* coinfection resulted in the presence of more neutrophils both in the early and the later stages of infection, times corresponding to the peaks of pathology due to *S*. *aureus* or *L*. *major*, respectively. Because of the paradoxical increase in the numbers of *S*. *aureus* in the face of elevated neutrophil numbers, we assessed neutrophil functions critical for antimicrobial action. At day 1 p.i., neutrophils recovered from *L*. *major-S*. *aureus* coinfected lesions phagocytosed either pathogen and had a functional NADPH oxidase, evidence suggesting that the antimicrobial machinery of recruited neutrophils was intact. Although both *S*. *aureus* and *L*. *major* can extend neutrophil lifespan [40, 63, 64], we found that a high percentage of neutrophils from *S*. *aureus-*containing lesions, either alone or with *L*. *major*, were apoptotic. In contrast, similar percentages of viable (*i*.*e*. AnnV^-^PI^-^) were recovered in all experimental conditions. Considering the markedly elevated number of total neutrophils in coinfected compared to singly infected groups, the net result was a large number of apoptotic neutrophils in *L*. *major-S*. *aureus* coinfected groups. Typically, apoptotic neutrophils are cleared by efferocytosis. However, expression of several transcripts associated with efferocytosis and wound healing, such as IL-10, IL-13, and MMP9, were downregulated at 3 days p.i. in *S*. *aureus* and coinfected ears. Taken together, these data demonstrate that neutrophils recruited to the sites of coinfection with *L*. *major-S*. *aureus* underwent apoptosis but were not cleared, thus contributing in part, to the large numbers of neutrophils in lesions but with failure to kill *S*. *aureus* or resolve the inflammation.

Although the mRNA transcript profiles for our selected chemokines/cytokines were similar between *L*. *major* singly or coinfected mouse ears at 28 days p.i., we found more IL-17A production from draining LN cells of coinfected mice in response to *L*. *major* antigen during coinfection. It is well established that acute *S*. *aureus* infection upregulates IL-17A, which contributes to the recruitment of neutrophils and consequently clearance of the pathogen [65]. Cutaneous bacteria can have a major impact on host immune status and the development of a Type 17 response to pathogens. Germ-free mice have fewer IL-17A-producing T cells at baseline and display less pathology in response to *L*. *major* infection despite higher parasite loads. In contrast, germ-free mice recolonized with *S*. *epidermidis* had restored numbers of IL-17A^+^ T cells in the skin and *L*. *major* lesions size similar to that of specific pathogen-free mice [4].

Th17 cells and IL-17A are also implicated in the immunopathology of murine models of CL [66–70]. Susceptible BALB/c mice have higher IL-17A levels in *L*. *major* lesions compared to resistant C57BL/6 mice [67]. Importantly, the levels of IL-17A produced by draining LN cells from our C57BL/6 four weeks post-single *L*. *major* infection group in response to whole promastigote *L*. *major* antigen were comparable to the levels they observed from the draining LN cells of *L*. *major-*infected C57BL/6 mice in response to soluble *Leishmania* antigen [67]. Although IL-17A has previously been implicated in CL, development of an elevated bystander Type 17 response to *L*. *major* in specific pathogen-free mice when coinfected with *S*. *aureus*, or the phenotypic consequences of such response, has not been described prior to the current study.

Bacterial-leishmanial coinfection is likely to occur in nature when an infected sand fly bites a mammalian host. When a sand fly takes a blood meal, it generates a pool of blood in the skin due to repeated probing activity. Bacteria from host skin, the sand fly midgut, and/or the environment may be deposited along with *Leishmania* spp. parasites in these dermal blood pools. Dey *et al*. recently demonstrated that culturable bacteria are deposited by sand flies transmitting *Leishmania donovani*, a cause of visceral leishmaniasis. The presence of bacteria contributes to the local priming and activation of the NLRP3 inflammasome, production of IL-1β, sustained recruitment of neutrophils, and enhanced dissemination of parasites to visceral organs [12]. NLRP3, ASC, or caspase-1/caspase-11 deficient BALB/c mice produced less IL-17A in *L*. *major-*infected footpads compared with wild-type mice [24]. Furthermore, elevated levels of IL-1β were detected in supernatants from cultured human *L*. *braziliensis* cutaneous leishmaniasis lesion biopsies compared to healthy skin controls, and a greater percentage of myeloid cells, particularly granulocytes, from lesion biopsies were pro-IL-1β^+^ compared to peripheral blood mononuclear cells from those same patients [21]. Thus, our observations may model the events occurring in human disease.

Our results suggest that *L*. *major* coinfection with *S*. *aureus* increases expression of pro-IL-1β and activation of IL-1β, which increases neutrophil infiltration, and stimulates γδ and non-γδ T cells in the skin to produce IL-17A. Interestingly, neutrophils, which have been implicated in the immunopathology of *L*. *major* lesions [23, 71], were a major cellular source of pro-IL-1β during coinfection. Both single infections with *S*. *aureus*, and double infection with both pathogens led to enhanced expression of IL-23 at 7 days p.i. IL-23 is important for the maintenance of Th17 cells [51, 52], and may contribute to the elevated IL-17A production in mice coinfected with *L*. *major* and *S*. *aureus* for four weeks.

A functional consequence of local IL-17A was suggested by antibody neutralization of IL-17A, a manipulation that partially ameliorated the exacerbated lesions observed during early coinfection. Interestingly, antibody neutralization of IL-1β did not mirror the results of anti-IL-17A treatment, but instead caused even further lesion exacerbation of coinfected lesions. This suggests that in the *L*. *major-S*. *aureus* coinfection context, IL-1β is important not only for promoting IL-17A production, but also the control of bacterial load. These data support a model in which *L*. *major* and *S*. *aureus* synergize to generate a microenvironment that promotes IL-1β and IL-23 production from myeloid cells, which contributes to IL-17A production by γδ and non-γδ T cells, the differentiation of naïve T cells into Th17 cells, and the continued recruitment of neutrophils. These neutrophils become apoptotic but are in an environment that delays their clearance and resolution of inflammation, which together, lead to increased lesion severity during coinfection.

**Fig 9:**
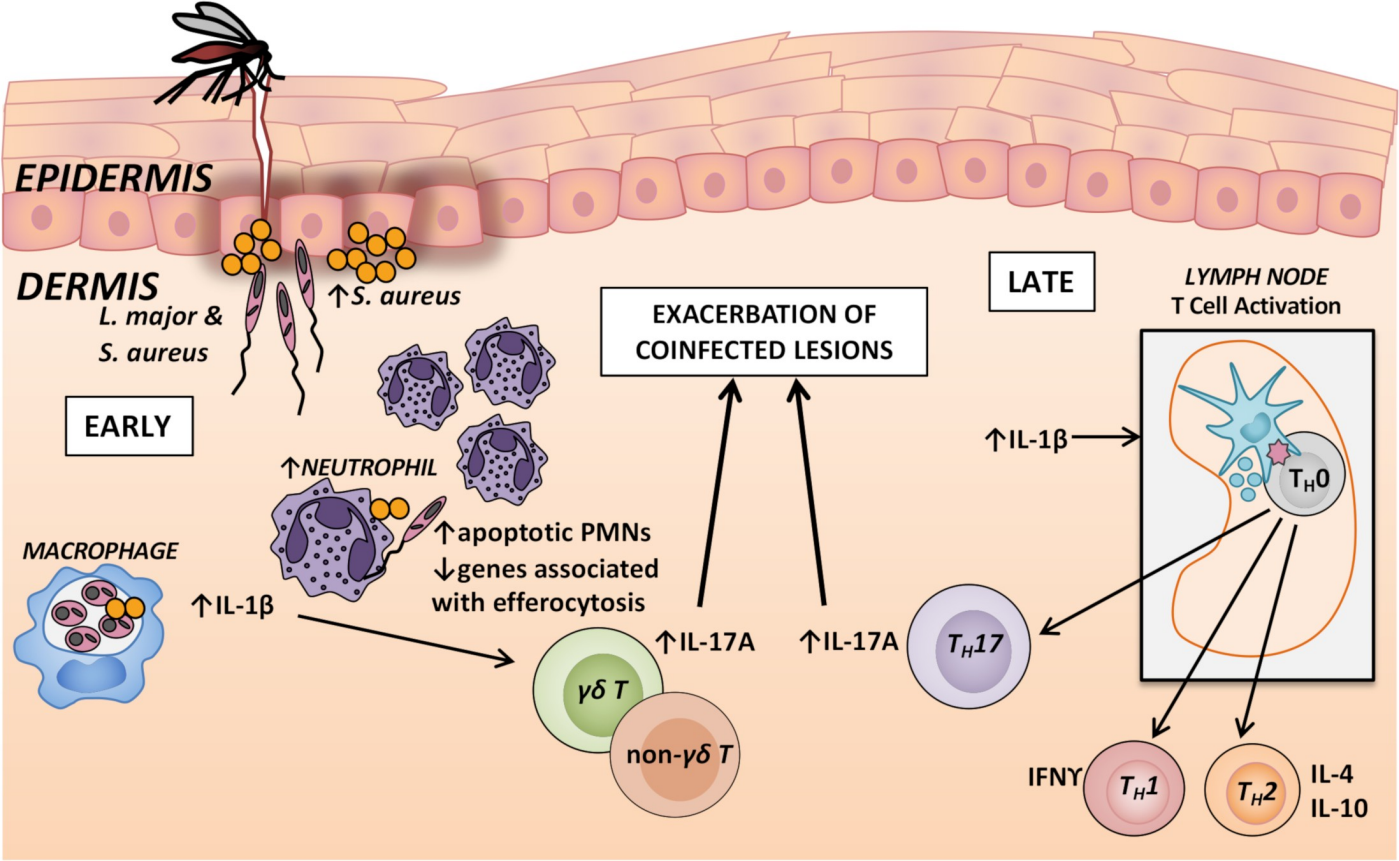
Proposed mechanism of *L*. *major* and *S*. *aureus* coinfection lesion exacerbation. When an infected sand fly takes a blood meal, it egests *L*. *major* into the skin, and *S*. *aureus* from the skin microbiota or from the sand fly gut also enters the skin. These organisms get taken up by host macrophages and neutrophils and promote the production of proinflammatory IL-1β and IL-23 by myeloid cells. The presence of *L*. *major* allows for enhanced *S*. *aureus* growth during the early stages of infection. Although it is reported that IL-1β and IL-23 promote the production of IL-17A from γδ and non-γδ T cells, and the differentiation of naïve T helper cells into Th17 cells, IL-1β may have an important role in control of *S*. *aureus* growth and lesion pathology related to bacterial burden. Elevated IL-17A expression in correlated with more neutrophils at the coinfected lesion site that were mostly apoptotic in a setting of downregulated efferocytosis. The IL-17A pathway may be largely responsible for the exacerbation of coinfected lesions.

In summary, we found that co-inoculation of *Staphylococcus aureu*s with *Leishmania major* at the same site in a murine skin infection model increased the *S*. *aureus* bacterial load but did not alter the local burden of the protozoan. Nonetheless, there was a local activation of IL-17A-mediated inflammatory responses and corresponding neutrophil recruitment to the site of coinfection, with exacerbation of both early and late phases of the inflammatory lesion. The large numbers of neutrophils recovered from the coinfections reflected increased recruitment due to IL-17A and decreased efferocytosis and clearance, though the molecular basis for this is not known. The exacerbated lesion size corresponded to an IL-17A response, which developed from an innate response of skin γδ and non-γδ T cells during the first week of infection, into an adaptive response of *L*. *major* antigen-responsive Th17 cells at later time points (Fig 9). These findings provide insight into the interactions that occur between *L*. *major* and microbiota from the sand fly midgut or mammalian host skin, and may contribute to the development of novel therapies that reduce immunopathology during cutaneous leishmaniasis.

## Supporting information

S1 Fig *S. aureus* inoculated in the ear at subclinical doses results in a slight increase in ear thickness. Mice ears were injected intradermally with 10^3^, 10^4^, 10^5^, 10^6^, or 10^7^ colony-forming units (CFUs) of *S*. *aureus* Newman and ear lesion length and width was measured over time, and area of the elliptical lesions were calculated. Data represent the mean ± SD of 2–5 mice/group.
(PDF)

S2 Fig *L. major* coinfection with *S. aureus* does not alter parasite burden during early phase of infection. Mice were injected intradermally in the ear with *L*. *major* (Lm) or *L*. *major* and *S*. *aureus* (L+S). Lm burden was measured by qPCR for *Leishmania* kinetoplastid DNA (kDNA) of DNA extracted from ears 3 and 7 days p.i. Data from one experiment with 5 mice/group. Error bars represent mean ± SD; ns = not significant by student’s *t-*test.
(PDF)

S3 Fig *L. major* coinfection with 10^3^ CFUs of *S. aureus* results in lesion exacerbation but no difference in parasite burden. (A) Mice were injected intradermally in the ear with PBS, Lm, Sa, or Lm+Sa and ear lesion volume was measured for 28 days. Asterisks (*) represent significance between Sa and coinfected groups. Crosshairs (#) represent *p*-value between Lm and coinfected groups. (B) Lm burden was measured by qPCR of DNA extracted from ear 28 days p.i. Data pooled from 3 separate experiments, each with 5 mice/group. Error bars represent mean ± SEM (A) or median and interquartile range (B). *p < 0.05, ** *p* < 0.01 two-way ANOVA with Tukey’s multiple comparisons test (A), ns = not significant by student’s *t-*test (B).
(PDF)

S4 Fig Gating strategies for neutrophil apoptosis and NADPH oxidase activity assays. (A) Neutrophil gating strategy for Annexin V and propidium iodide (PI) to assess apoptosis. (B) Neutrophil gating strategy for dihydrorhodamine 123 (DHR) to assess phagocyte NADPH oxidase activity. (C) Histogram plot of unstimulated DHR-added cells from a phosphate buffer saline injected mouse to represent the fluorescence minus one used to determine the DHR gate.
(PDF)

S5 Fig Inoculation of 10^4^ CFUs of *S. aureus* in singly and coinfected ears resulted in resulted in downregulation of proinflammatory and efferocytosis related genes at 3 days post-infection. Ears were harvested, RNA extracted, and cDNA made and pre-amplified. Samples and Taqman gene expression assays were loaded onto a 48x48 Fluidigm dynamic array. C_T_ values were normalized to GUSB and to the average value of the PBS group for each assay to get the -ΔΔC_T_, yielding the log_2_(fold change). Each data point represents one mouse. Data represent the mean ± SD of one experiment with 4–5 mice/group. *p < 0.05, **p < 0.01 by one-way ANOVA with Tukey’s multiple comparisons test.
(PDF)

S6 Fig Inflammatory gene expression is similar between *L. major* and *L. major-S*. aureus coinfected ears at 28 days post-infection. Ears were harvested, RNA extracted, and cDNA made and pre-amplified. Samples and Taqman gene expression assays were loaded onto a 48x48 Fluidigm dynamic array. C_T_ values were normalized to GAPDH and to the average value of the PBS group for each assay to get the -ΔΔC_T_, yielding the log_2_(fold change). Each data point represents one mouse. Data represent the mean ± SEM of 3 pooled experiments, each with 4–5 mice/group. *p < 0.05, **p < 0.01 by one-way ANOVA with Tukey’s multiple comparisons test.
(PDF)

S7 Fig Gating strategy for lymphoid surface staining and intracellular cytokine stains. Cells were gated by forward scatter (FSC) x side scatter (SSC) followed by FSC x FSC-Width to obtain single cells. CD45 was used as a marker of hematopoietic cells, followed by Thy1.2 for T cells. T cells were further delineated by expression of γδ T cell receptor, and expression of IL-17A or IFNγ. Fluorescence minus one (FMO) controls were used to gate on cells positive for expression of IL-17A or IFNγ.
(PDF)

S8 Fig Gating strategy for myeloid surface stains and IL-17A intracellular cytokine stain. Cells were gated by forward scatter (FSC) x side scatter (SSC) followed by FSC x FSC-Width to obtain single cells. CD45 was used as a marker of hematopoietic cells, followed by Thy1.2 to exclude T cells, and CD11b as a marker expressed by myeloid cells. Dendritic cells (DC) were defined as CD45^+^ CD11b^+^ CD11c^+^ cells. CD11b^+^ were further delineated by expression of Ly6G and Ly6C. Neutrophils (PMN) were defined as CD45^+^ CD11b^+^ Ly6G^hi^ Ly6C^int^, and inflammatory monocytes (MN) were defined as CD45^+^ CD11b^+^ Ly6G^-^ Ly6C^hi^. Fluorescence minus one (FMO) controls were used to gate on cells positive for expression of IL-17A.
(PDF)

S9 Fig Gating strategy for myeloid surface stains and IL-1β intracellular cytokine stain. Cells were gated by forward scatter (FSC) x side scatter (SSC) followed by FSC x FSC-Width to obtain single cells. CD45 was used as a marker of hematopoietic cells, followed by CD11b as a marker of myeloid cells. Dendritic cells (DC) were defined as CD45^+^ CD11b^+^ CD11c^+^ cells. Other CD11b^+^ cells were further delineated by expression of Ly6G and Ly6C. Neutrophils (PMN) were defined as CD45^+^ CD11b^+^ Ly6G^hi^ Ly6C^int^, and inflammatory monocytes (MN) were defined as CD45^+^ CD11b^+^ Ly6G^-^ Ly6C^hi^. Fluorescence minus one (FMO) controls were used to gate on cells positive for expression of IL-1β.
(PDF)

S10 Fig Treatment with anti-IL-1β neutralizing antibodies reduces but does not deplete IL-1β in mouse ears. In order to confirm the efficacy of anti-IL-1β antibodies, mice were injected intraperitoneally with polyclonal IgG antibodies (isotype), anti-IL-1β antibodies (α-IL-1β), no antibodies (No IgG), and then injected in the right-sided ear with 5x10^5^ colony-forming units of *S*. *aureus* Newman as a strong stimulus for IL-1β release. On day 1 p.i. ears were snap frozen in liquid nitrogen and subsequently homogenized in cell/tissue lysis buffer and assayed in an IL-1β ELISA to determine IL-1β concentrations. Data are shown as the mean ± SD of one experiment with 1–2 mice/group.
(PDF)

S1 Table Inflammatory gene expression between *S. aureus, L. major*, and *L. major-S. aureus* coinfected ears at different doses at 28 days post-infection. C_T_ values were normalized to GAPDH and to the average value of the PBS group for each assay to get the -ΔΔC_T_, yielding the log_2_(fold change). Data shown as the mean ± SEM of three independent experiments, each with 4–5 mice/group.
(PDF)
