# Supplementary material for: Coinfection with Leishmania major and Staphylococcus aureus enhances the pathologic responses to both microbes through a pathway involving IL-17A
Source: PLoS Negl Trop Dis. 2019 May 20;13(5):e0007247. doi: 10.1371/journal.pntd.0007247 (PMC6527190; doi:10.1371/journal.pntd.0007247)
Supplement: S1 Fig — Mice ears were injected intradermally with 103, 104, 105, 106, or 107 colony-forming units (CFUs) of S. aureus Newman and ear lesion length and width was measured over time, and area of the elliptical lesions were calculated. Data represent the mean ± SD of 2–5 mice/group. (PDF) [file pntd.0007247.s001.pdf]

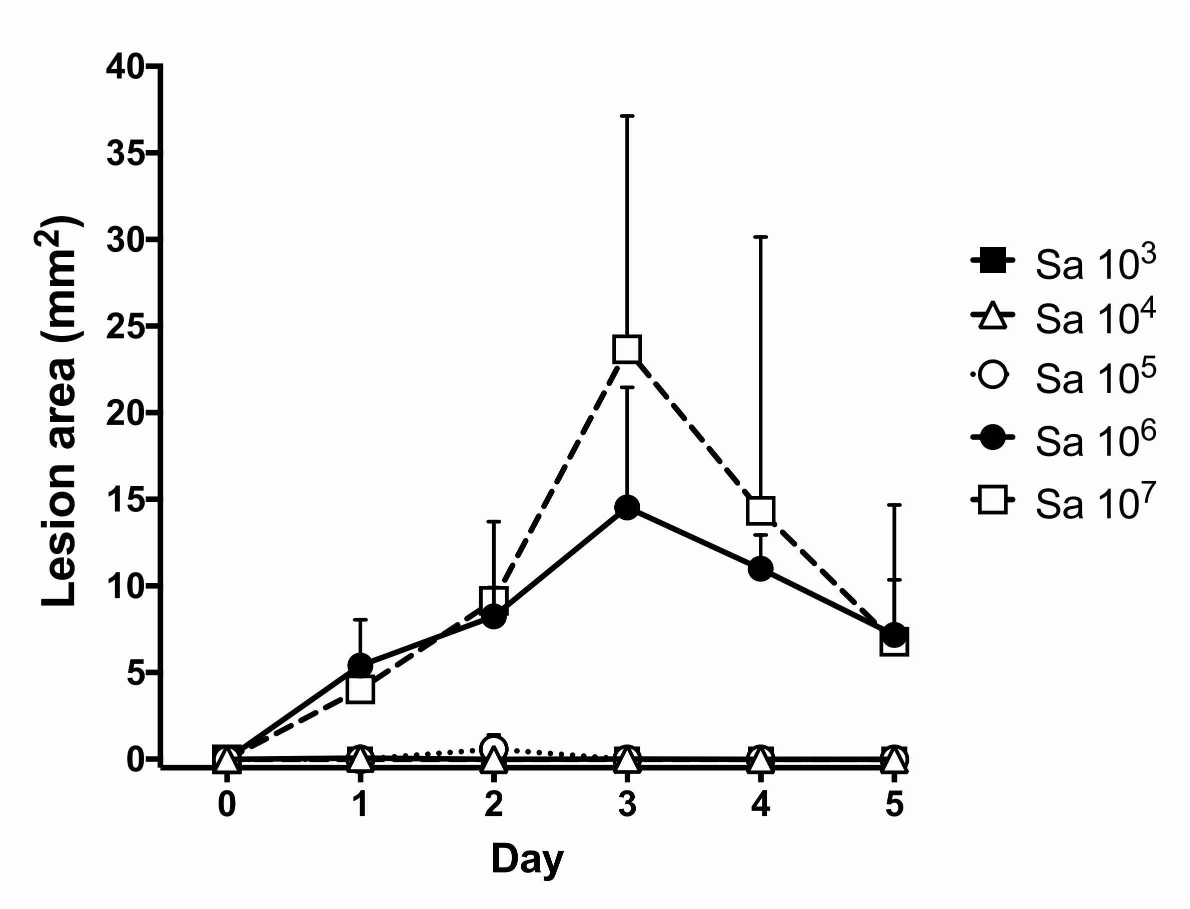


**S1 Figure. *S. aureus* inoculated in the ear at subclinical doses results in a slight increase in ear thickness.** Mice ears were injected intradermally with $10^3$, $10^4$, $10^5$, $10^6$, or $10^7$ colony-forming units (CFUs) of *S. aureus* Newman and ear lesion length and width was measured over time, and area of the elliptical lesions were calculated. Data represent the mean ± SD of 2-5 mice/group.
