# Supplementary material for: Coinfection with Leishmania major and Staphylococcus aureus enhances the pathologic responses to both microbes through a pathway involving IL-17A
Source: PLoS Negl Trop Dis. 2019 May 20;13(5):e0007247. doi: 10.1371/journal.pntd.0007247 (PMC6527190; doi:10.1371/journal.pntd.0007247)
Supplement: S2 Fig — Mice were injected intradermally in the ear with L. major (Lm) or L. major and S. aureus (L+S). Lm burden was measured by qPCR for Leishmania kinetoplastid DNA (kDNA) of DNA extracted from ears 3 and 7 days p.i. Data from one experiment with 5 mice/group. Error bars represent mean ± SD; ns = not significant by student’s t-test. (PDF) [file pntd.0007247.s002.pdf]

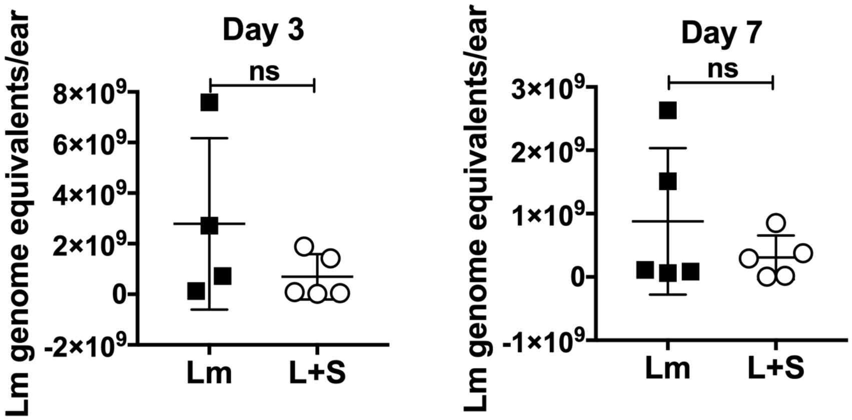

S2 Figure. *L. major* coinfection with *S. aureus* does not alter parasite burden during early phase of infection. Mice were injected intradermally in the ear with *L. major* (Lm) or *L. major* and *S. aureus* (L+S). Lm burden was measured by qPCR for *Leishmania* kinetoplastid DNA (kDNA) of DNA extracted from ears 3 and 7 days p.i. Data from one experiment with 5 mice/group. Error bars represent mean ± SD; ns = not significant by student's *t*-test.
