# Supplementary material for: Coinfection with Leishmania major and Staphylococcus aureus enhances the pathologic responses to both microbes through a pathway involving IL-17A
Source: PLoS Negl Trop Dis. 2019 May 20;13(5):e0007247. doi: 10.1371/journal.pntd.0007247 (PMC6527190; doi:10.1371/journal.pntd.0007247)
Supplement: S3 Fig — (A) Mice were injected intradermally in the ear with PBS, Lm, Sa, or Lm+Sa and ear lesion volume was measured for 28 days. Asterisks (*) represent significance between Sa and coinfected groups. Crosshairs (#) represent p-value between Lm and coinfected groups. (B) Lm burden was measured by qPCR of DNA extracted from ear 28 days p.i. Data pooled from 3 separate experiments, each with 5 mice/group. Error bars represent mean ± SEM (A) or median and interquartile range (B). *p < 0.05, ** p < 0.01 two-way ANOVA with Tukey’s multiple comparisons test (A), ns = not significant by student’s t-test (B). (PDF) [file pntd.0007247.s003.pdf]

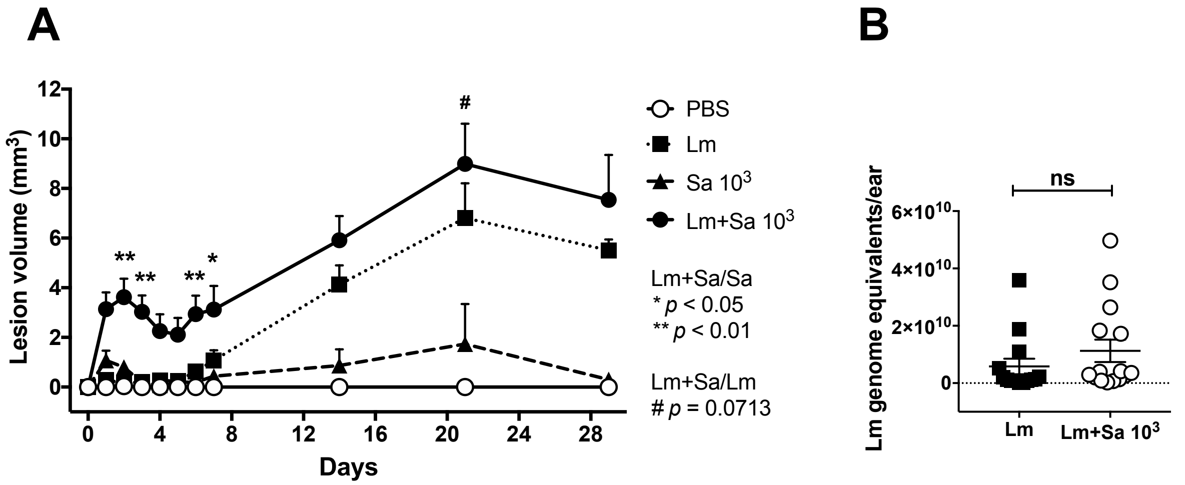


**S3 Figure. *L. major* coinfection with $10^3$ CFUs of *S. aureus* results in lesion exacerbation but no difference in parasite burden.** (A) Mice were injected intradermally in the ear with PBS, Lm, Sa, or Lm+Sa and ear lesion volume was measured for 28 days. Asterisks (*) represent significance between Sa and coinfected groups. Crosshairs (#) represent *p*-value between Lm and coinfected groups. (B) Lm burden was measured by qPCR of DNA extracted from ear 28 days p.i. Data pooled from 3 separate experiments, each with 5 mice/group. Error bars represent mean ± SEM (A) or median and interquartile range (B). *$p < 0.05$, ** $p < 0.01$ two-way ANOVA with Tukey's multiple comparisons test (A), ns = not significant by student's *t*-test (B).
