# Supplementary material for: Coinfection with Leishmania major and Staphylococcus aureus enhances the pathologic responses to both microbes through a pathway involving IL-17A
Source: PLoS Negl Trop Dis. 2019 May 20;13(5):e0007247. doi: 10.1371/journal.pntd.0007247 (PMC6527190; doi:10.1371/journal.pntd.0007247)
Supplement: S4 Fig — (A) Neutrophil gating strategy for Annexin V and propidium iodide (PI) to assess apoptosis. (B) Neutrophil gating strategy for dihydrorhodamine 123 (DHR) to assess phagocyte NADPH oxidase activity. (C) Histogram plot of unstimulated DHR-added cells from a phosphate buffer saline injected mouse to represent the fluorescence minus one used to determine the DHR gate. (PDF) [file pntd.0007247.s004.pdf]

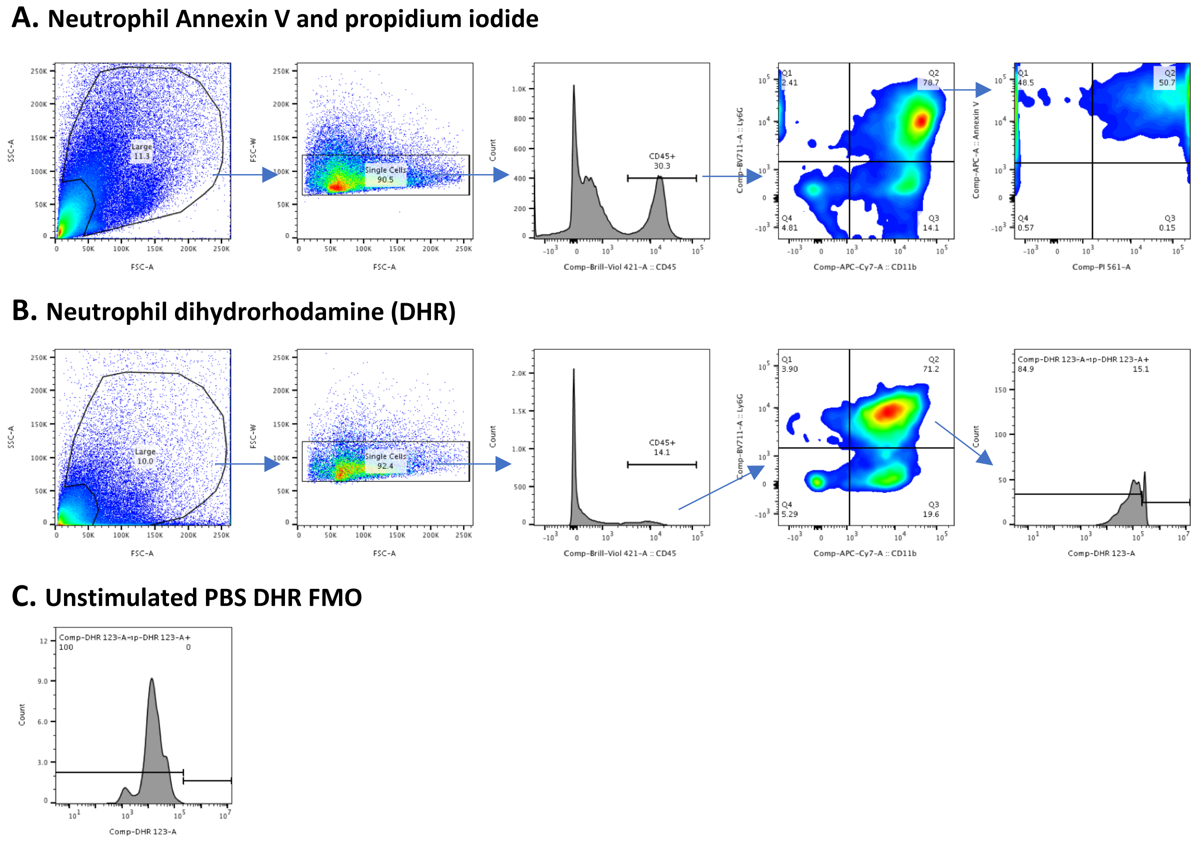

S4 Figure. Gating strategies for neutrophil apoptosis and NADPH oxidase activity assays. (A) Neutrophil gating strategy for Annexin V and propidium iodide (PI) to assess apoptosis. (B) Neutrophil gating strategy for dihydrorhodamine 123 (DHR) to assess NADPH oxidase activity in response to PMA. (C) Histogram plot of unstimulated DHR-added cells from a phosphate buffer saline injected mouse to represent the fluorescence minus one used to determine the DHR gate.
