# Supplementary material for: Coinfection with Leishmania major and Staphylococcus aureus enhances the pathologic responses to both microbes through a pathway involving IL-17A
Source: PLoS Negl Trop Dis. 2019 May 20;13(5):e0007247. doi: 10.1371/journal.pntd.0007247 (PMC6527190; doi:10.1371/journal.pntd.0007247)
Supplement: S5 Fig — Ears were harvested, RNA extracted, and cDNA made and pre-amplified. Samples and Taqman gene expression assays were loaded onto a 48x48 Fluidigm dynamic array. CT values were normalized to GUSB and to the average value of the PBS group for each assay to get the -ΔΔCT, yielding the log2(fold change). Each data point represents one mouse. Data represent the mean ± SD of one experiment with 4–5 mice/group. *p < 0.05, **p < 0.01 by one-way ANOVA with Tukey’s multiple comparisons test. (PDF) [file pntd.0007247.s005.pdf]

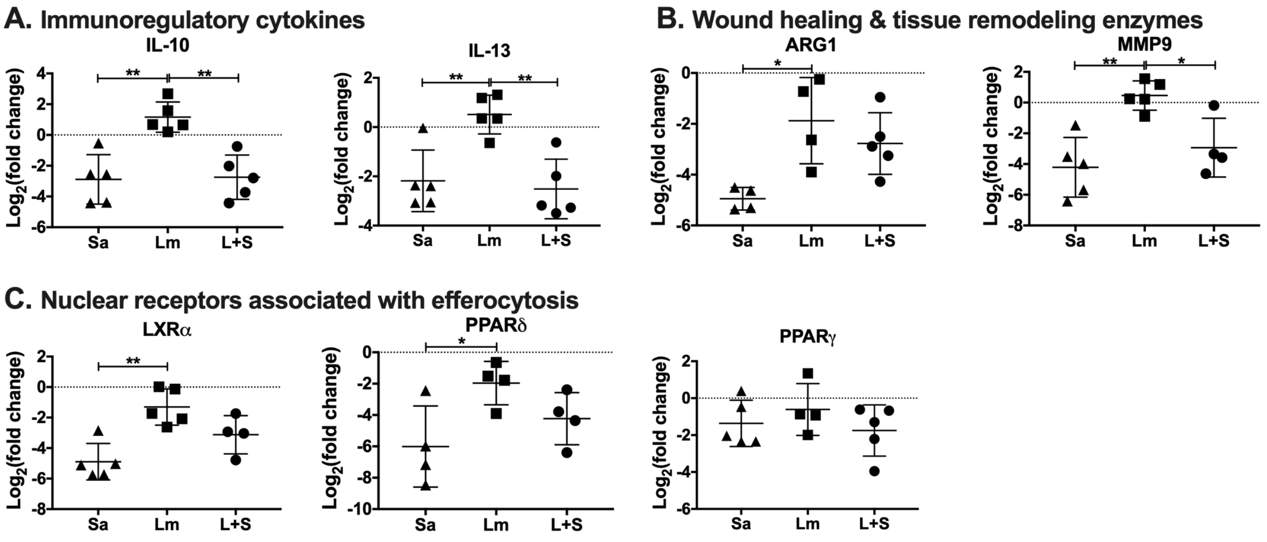

S5 Figure. Inoculation of 10^4 colony-forming units of *S. aureus* in singly and coinfected ears resulted in resulted in downregulation of proinflammatory and efferocytosis related genes at 3 days post-infection. Ears were harvested, RNA extracted, and cDNA made and pre-amplified. Samples and Taqman gene expression assays were loaded onto a 48x48 Fluidigm dynamic array. C_T values were normalized to GUSB and to the average value of the PBS group for each assay to get the -$\Delta\Delta C_T$, yielding the $\log_2$(fold change). Each data point represents one mouse. Data represent the mean ± SD of one experiment with 4-5 mice/group. *$p < 0.05$, **$p < 0.01$ by one-way ANOVA with Tukey's multiple comparisons test.
