# Supplementary material for: Coinfection with Leishmania major and Staphylococcus aureus enhances the pathologic responses to both microbes through a pathway involving IL-17A
Source: PLoS Negl Trop Dis. 2019 May 20;13(5):e0007247. doi: 10.1371/journal.pntd.0007247 (PMC6527190; doi:10.1371/journal.pntd.0007247)
Supplement: S6 Fig — Ears were harvested, RNA extracted, and cDNA made and pre-amplified. Samples and Taqman gene expression assays were loaded onto a 48x48 Fluidigm dynamic array. CT values were normalized to GAPDH and to the average value of the PBS group for each assay to get the -ΔΔCT, yielding the log2(fold change). Each data point represents one mouse. Data represent the mean ± SEM of 3 pooled experiments, each with 4–5 mice/group. *p < 0.05, **p < 0.01 by one-way ANOVA with Tukey’s multiple comparisons test. (PDF) [file pntd.0007247.s006.pdf]

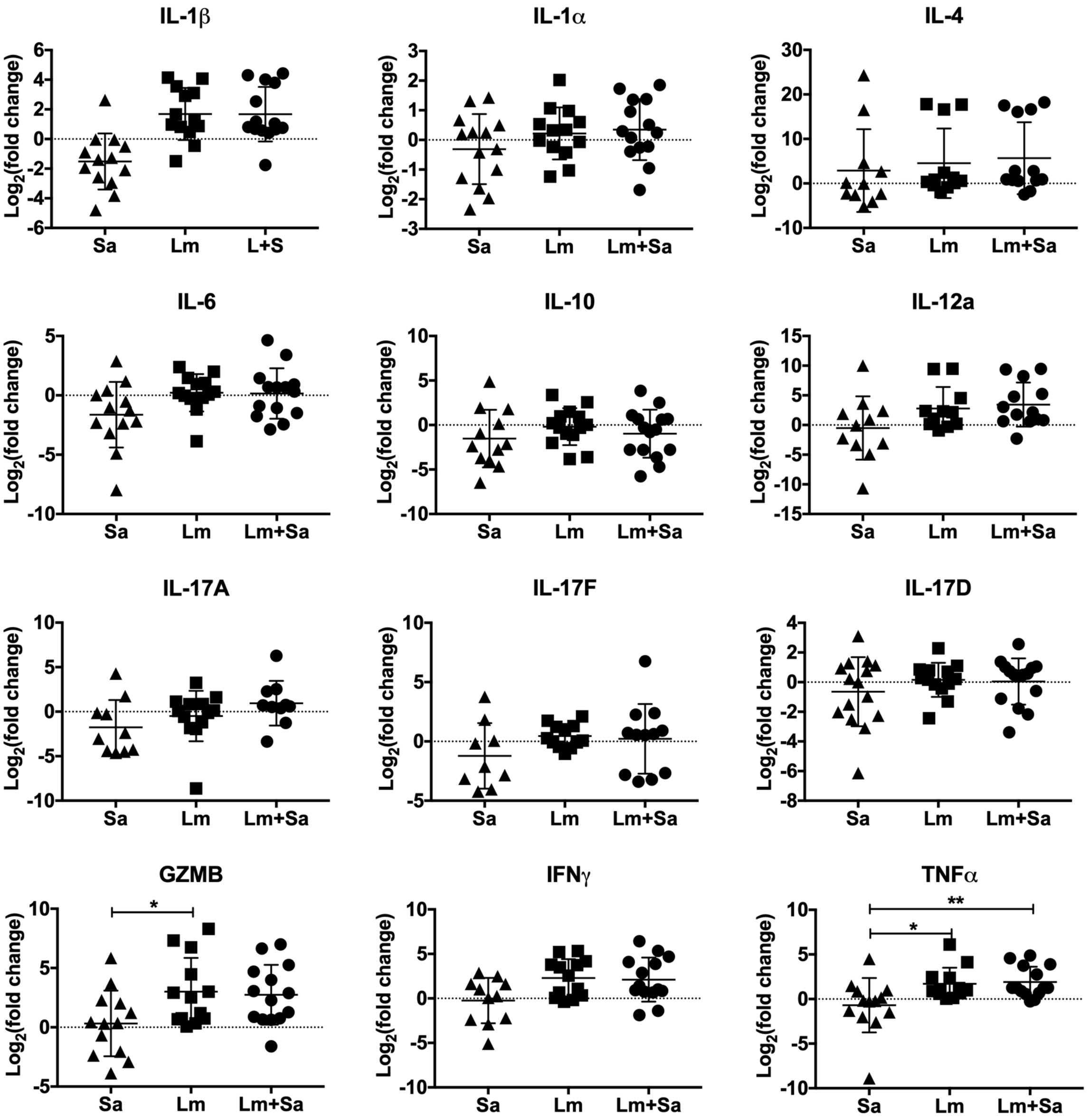

S6 Figure. Inflammatory gene expression is similar between *L. major* and *L. major-S. aureus* coinfected ears at 28 days post-infection. Ears were harvested, RNA extracted, and cDNA made and pre-amplified. Samples and Taqman gene expression assays were loaded onto a 48x48 Fluidigm dynamic array. C_T values were normalized to GAPDH and to the average value of the PBS group for each assay to get the -$\Delta\Delta C_T$, yielding the $\log_2$(fold change). Each data point represents one mouse. Data represent the mean ± SEM of 3 pooled experiments, each with 4-5 mice/group. *p < 0.05, **p < 0.01 by one-way ANOVA with Tukey's multiple comparisons test.
