# Supplementary material for: Coinfection with Leishmania major and Staphylococcus aureus enhances the pathologic responses to both microbes through a pathway involving IL-17A
Source: PLoS Negl Trop Dis. 2019 May 20;13(5):e0007247. doi: 10.1371/journal.pntd.0007247 (PMC6527190; doi:10.1371/journal.pntd.0007247)
Supplement: S7 Fig — Cells were gated by forward scatter (FSC) x side scatter (SSC) followed by FSC x FSC-Width to obtain single cells. CD45 was used as a marker of hematopoietic cells, followed by Thy1.2 for T cells. T cells were further delineated by expression of γδ T cell receptor, and expression of IL-17A or IFNγ. Fluorescence minus one (FMO) controls were used to gate on cells positive for expression of IL-17A or IFNγ. (PDF) [file pntd.0007247.s007.pdf]

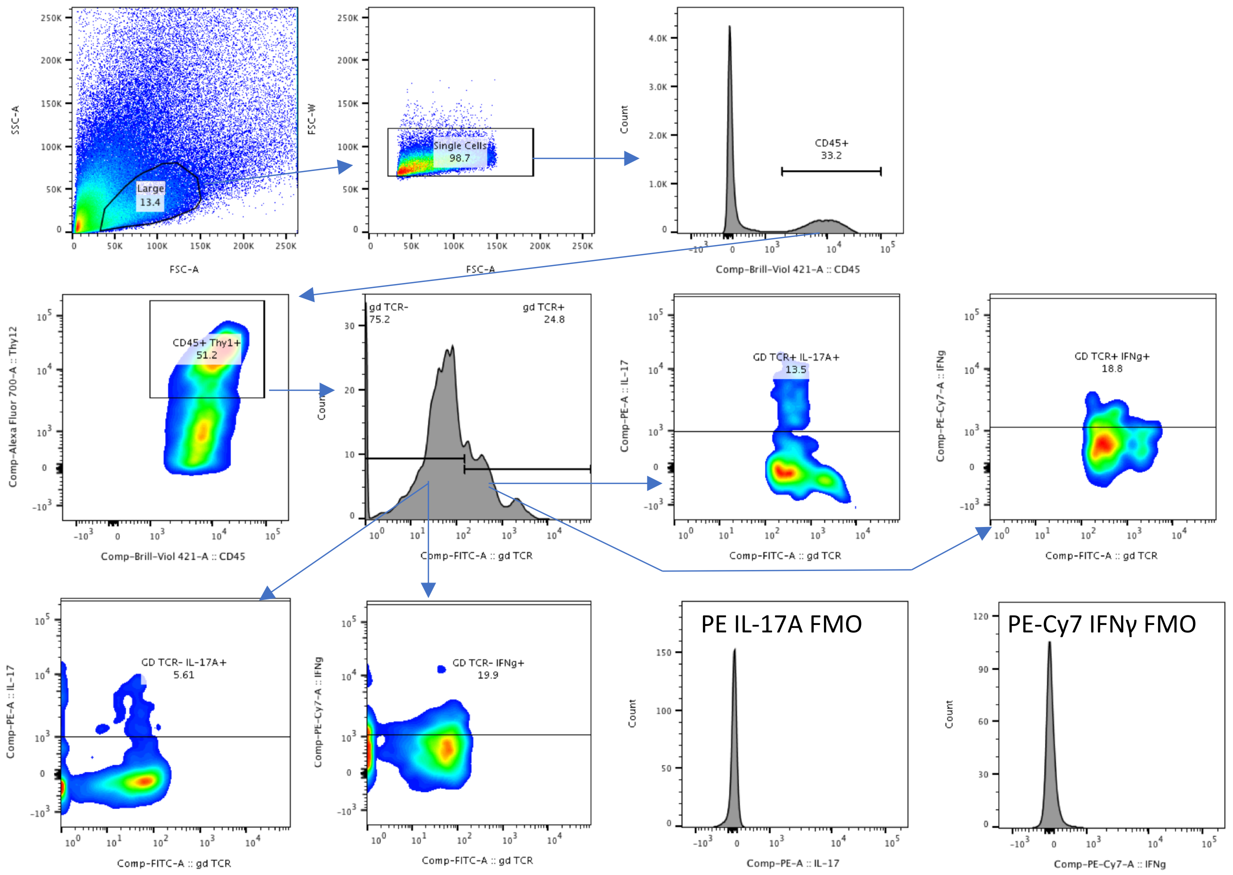

S7 Figure. Gating strategy for lymphoid surface staining and intracellular cytokine stains. Cells were gated by forward scatter (FSC) x side scatter (SSC) followed by FSC x FSC-Width to obtain single cells. CD45 was used as a marker of hematopoietic cells, followed by Thy1.2 for T cells. T cells were further delineated by expression of γδ T cell receptor, and expression of IL-17A or IFNγ. Fluorescence minus one (FMO) controls were used to gate on cells positive for expression of IL-17A or IFNγ.
