# Supplementary material for: Coinfection with Leishmania major and Staphylococcus aureus enhances the pathologic responses to both microbes through a pathway involving IL-17A
Source: PLoS Negl Trop Dis. 2019 May 20;13(5):e0007247. doi: 10.1371/journal.pntd.0007247 (PMC6527190; doi:10.1371/journal.pntd.0007247)
Supplement: S8 Fig — Cells were gated by forward scatter (FSC) x side scatter (SSC) followed by FSC x FSC-Width to obtain single cells. CD45 was used as a marker of hematopoietic cells, followed by Thy1.2 to exclude T cells, and CD11b as a marker expressed by myeloid cells. Dendritic cells (DC) were defined as CD45+ CD11b+ CD11c+ cells. CD11b+ were further delineated by expression of Ly6G and Ly6C. Neutrophils (PMN) were defined as CD45+ CD11b+ Ly6Ghi Ly6Cint, and inflammatory monocytes (MN) were defined as CD45+ CD11b+ Ly6G- Ly6Chi. Fluorescence minus one (FMO) controls were used to gate on cells positive for expression of IL-17A. (PDF) [file pntd.0007247.s008.pdf]

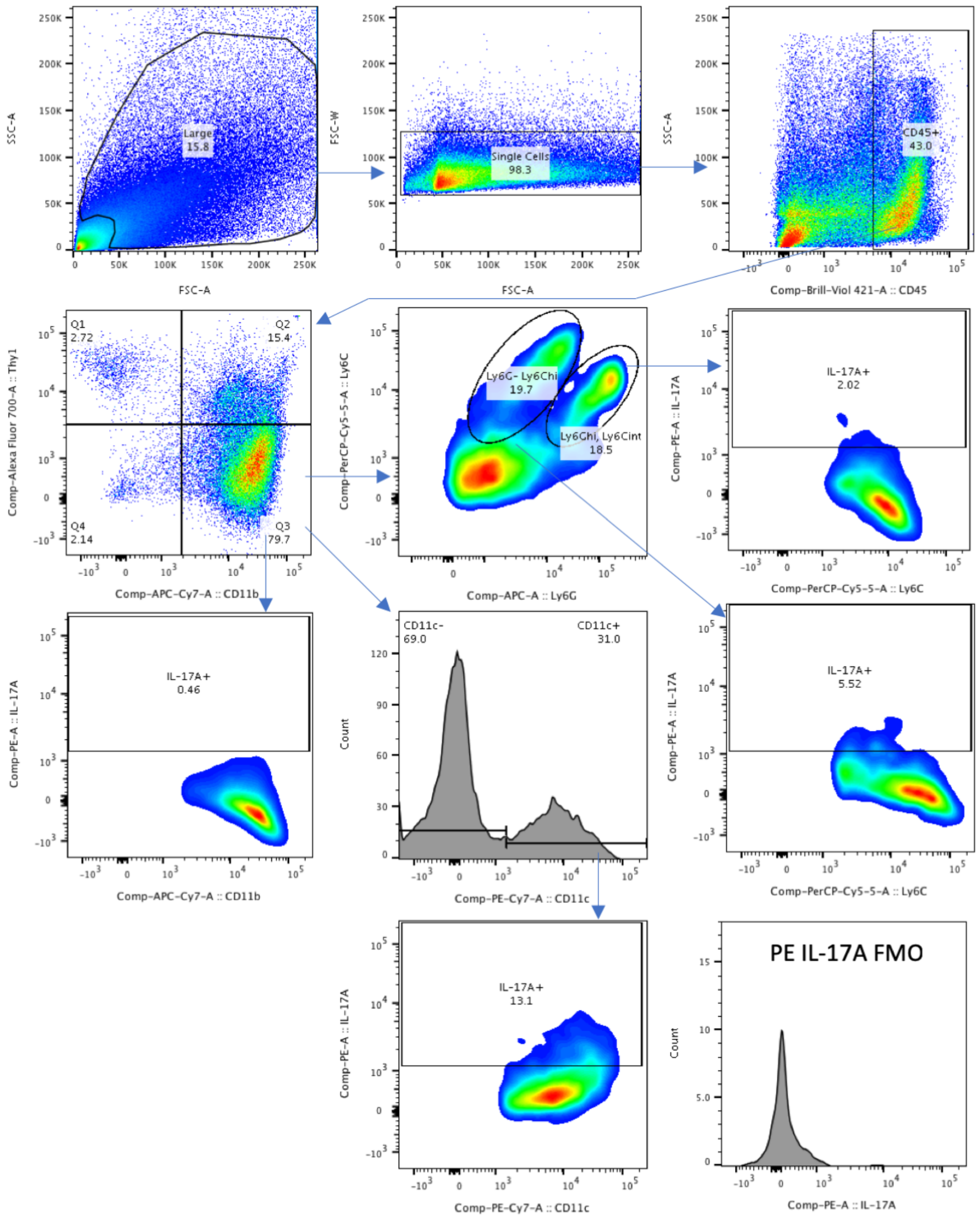

S8 Figure. Gating strategy for myeloid surface staining and intracellular cytokine stains. Cells were gated by forward scatter (FSC) x side scatter (SSC) followed by FSC x FSC-Width to obtain single cells. CD45 was used as a marker of hematopoietic cells, followed by Thy1.2 to exclude T cells, and CD11b as a marker expressed by myeloid cells. Dendritic cells (DC) were defined as $CD45^+$ $CD11b^+$ $CD11c^+$ cells. $CD11b^+$ were further delineated by expression of Ly6G and Ly6C. Neutrophils (PMN) were defined as $CD45^+$ $CD11b^+$ $Ly6G^{hi}$ $Ly6C^{int}$, and inflammatory monocytes (MN) were defined as $CD45^+$ $CD11b^+$ $Ly6G^-$ $Ly6C^{hi}$. Fluorescence minus one (FMO) controls were used to gate on cells positive for expression of IL-17A.
