# Supplementary material for: Coinfection with Leishmania major and Staphylococcus aureus enhances the pathologic responses to both microbes through a pathway involving IL-17A
Source: PLoS Negl Trop Dis. 2019 May 20;13(5):e0007247. doi: 10.1371/journal.pntd.0007247 (PMC6527190; doi:10.1371/journal.pntd.0007247)
Supplement: S9 Fig — Cells were gated by forward scatter (FSC) x side scatter (SSC) followed by FSC x FSC-Width to obtain single cells. CD45 was used as a marker of hematopoietic cells, followed by CD11b as a marker of myeloid cells. Dendritic cells (DC) were defined as CD45+ CD11b+ CD11c+ cells. Other CD11b+ cells were further delineated by expression of Ly6G and Ly6C. Neutrophils (PMN) were defined as CD45+ CD11b+ Ly6Ghi Ly6Cint, and inflammatory monocytes (MN) were defined as CD45+ CD11b+ Ly6G- Ly6Chi. Fluorescence minus one (FMO) controls were used to gate on cells positive for expression of IL-1β. (PDF) [file pntd.0007247.s009.pdf]

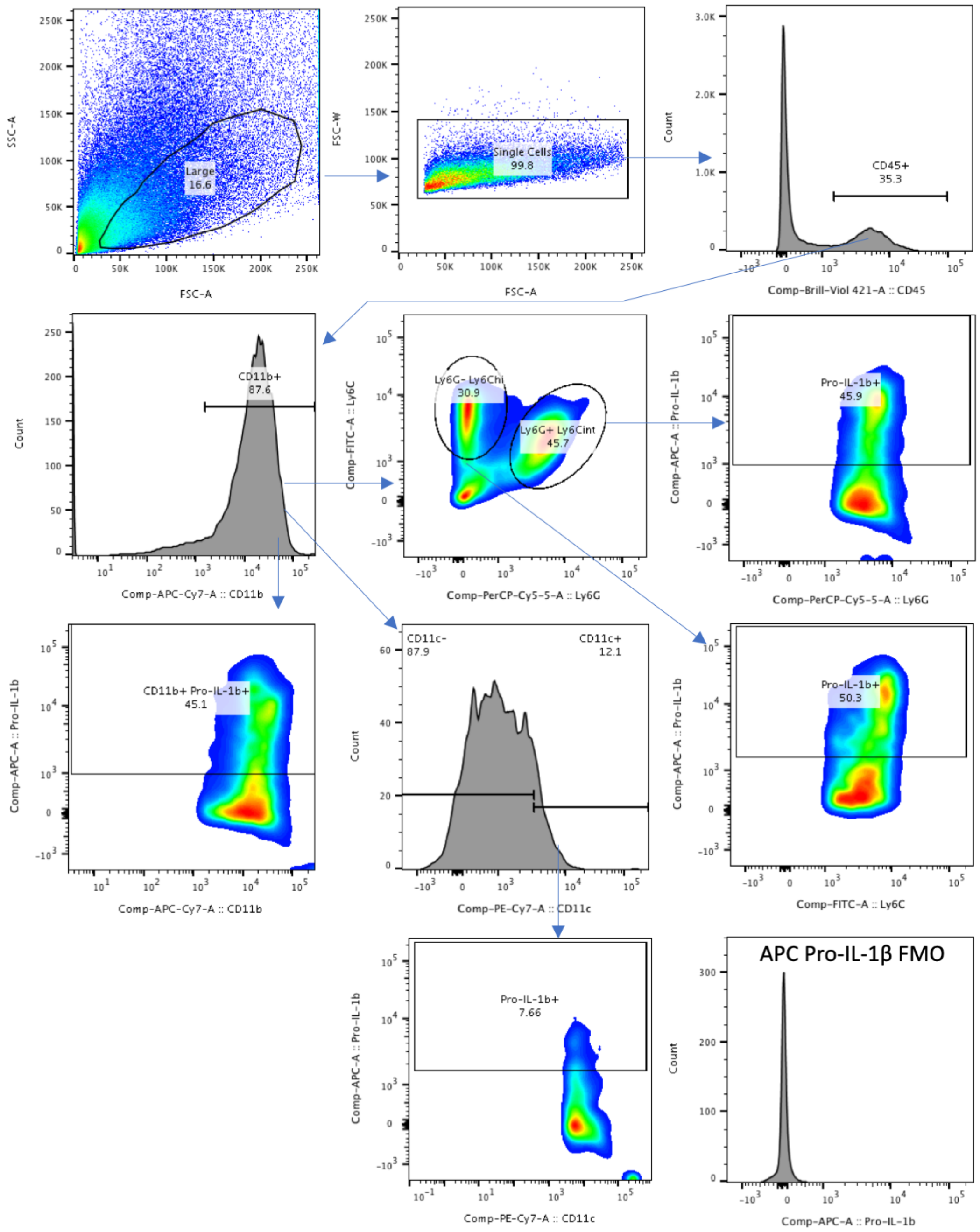

S9 Figure. Gating strategy for myeloid surface stains and IL-1β intracellular cytokine stain. Cells were gated by forward scatter (FSC) x side scatter (SSC) followed by FSC x FSC-Width to obtain single cells. CD45 was used as a marker of hematopoietic cells, followed by CD11b as a marker of myeloid cells. Dendritic cells (DC) were defined as $CD45^{+}$ $CD11b^{+}$ $CD11c^{+}$ cells. Other $CD11b^{+}$ cells were further delineated by expression of Ly6G and Ly6C. Neutrophils (PMN) were defined as $CD45^{+}$ $CD11b^{+}$ $Ly6G^{hi}$ $Ly6C^{int}$, and inflammatory monocytes (MN) were defined as $CD45^{+}$ $CD11b^{+}$ $Ly6G^{-}$ $Ly6C^{hi}$. Fluorescence minus one (FMO) controls were used to gate on cells positive for expression of IL-1β.
