# Supplementary material for: Coinfection with Leishmania major and Staphylococcus aureus enhances the pathologic responses to both microbes through a pathway involving IL-17A
Source: PLoS Negl Trop Dis. 2019 May 20;13(5):e0007247. doi: 10.1371/journal.pntd.0007247 (PMC6527190; doi:10.1371/journal.pntd.0007247)
Supplement: S10 Fig — In order to confirm the efficacy of anti-IL-1β antibodies, mice were injected intraperitoneally with polyclonal IgG antibodies (isotype), anti-IL-1β antibodies (α-IL-1β), no antibodies (No IgG), and then injected in the right-sided ear with 5x10^5^ colony-forming units of S. aureus Newman as a strong stimulus for IL-1β release. On day 1 p.i. ears were snap frozen in liquid nitrogen and subsequently homogenized in cell/tissue lysis buffer and assayed in an IL-1β ELISA to determine IL-1β concentrations. Data are shown as the mean ± SD of one experiment with 1–2 mice/group. (PDF) [file pntd.0007247.s010.pdf]

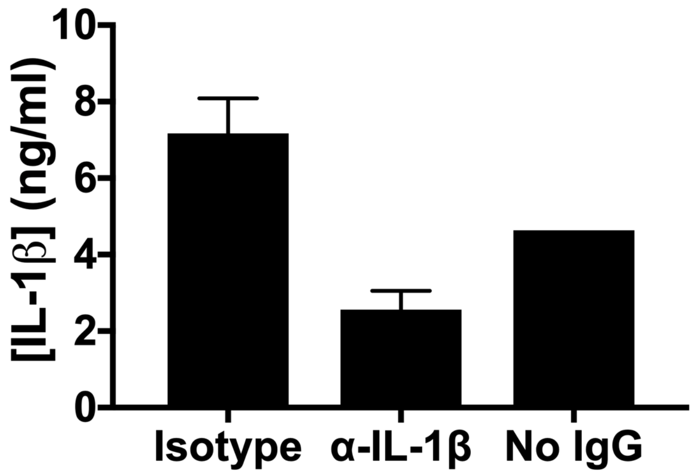

S10 Figure. Treatment with anti-IL-1β neutralizing antibodies reduces but does not deplete IL-1β in mouse ears. In order to confirm the efficacy of anti-IL-1β antibodies, mice were injected intraperitoneally with polyclonal IgG antibodies (isotype), anti-IL-1β antibodies (α-IL-1β), no antibodies (No IgG), and then injected in the right-sided ear with $5x10^5$ colony-forming units of *S. aureus* Newman as a strong stimulus for IL-1β release. On day 1 p.i. ears were snap frozen in liquid nitrogen and subsequently homogenized in cell/tissue lysis buffer and assayed in an IL-1β ELISA to determine IL-1β concentrations. Data are shown as the mean ± SD of one experiment with 1-2 mice/group.
