# Supplementary material for: Coinfection with Leishmania major and Staphylococcus aureus enhances the pathologic responses to both microbes through a pathway involving IL-17A
Source: PLoS Negl Trop Dis. 2019 May 20;13(5):e0007247. doi: 10.1371/journal.pntd.0007247 (PMC6527190; doi:10.1371/journal.pntd.0007247)
Supplement: S1 Table — CT values were normalized to GAPDH and to the average value of the PBS group for each assay to get the -ΔΔCT, yielding the log2(fold change). Data shown as the mean ± SEM of three independent experiments, each with 4–5 mice/group. (PDF) [file pntd.0007247.s011.pdf]

	Sa 10^3	Sa 10^4	Lm 10^6	Lm 10^6+Sa 10^3	Lm 10^6+Sa 10^4
**IL-1α**	-1.02 ± 0.61	-0.28 ± 0.62	0.26 ± 0.38	0.20 ± 0.41	0.29 ± 0.59
**IL-1β**	-.243 ± 0.63	-1.59 ± 0.43	0.26 ± 0.37	0.199 ± 0.41	1.73 ± 0.71
**IL-1rn**	-1.16 ± 0.60	-0.31 ± 0.47	0.23 ± 0.30	0.04 ± 0.44	0.56 ± 0.32
**NLRP10**	-1.85 ± 1.08	-0.47 ± 0.49	-0.63 ± 0.89	-0.30 ± 0.79	-0.30 ± 0.46
**NLRP12**	-4.07 ± 1.88	-2.65 ± 2.11	-2.34 ± 2.26	-1.26 ± 2.54	-1.99 ± 2.33
**TGFBR1**	-1.00 ± 0.83	0.17 ± 1.09	0.60 ± 0.40	0.91 ± 0.35	0.85 ± 0.47
**TGFBR2**	-0.80 ± 0.59	0.27 ± 0.59	0.02 ± 0.28	0.10 ± 0.33	0.48 ±0.24
**EMR1**	-1.01 ± 0.53	0.05 ± 0.15	1.14 ± 0.17	1.26 ± 0.66	1.24 ± 0.17
**CSF1R**	-0.41 ± 0.24	0.68 ± 0.61	1.34 ± 0.25	1.12 ± 0.68	1.42 ± 0.34
**GZMB**	-0.62 ± 1.43	0.80 ± 1.74	3.20 ± 1.67	3.43 ± 1.51	2.93 ± 1.51
**CD3e**	-0.91 ± 0.46	0.35 ± 0.87	1.08 ± 0.53	1.27 ± 0.49	1.11 ± 0.52
**CD86**	-0.28 ± 0.39	0.85 ± 0.97	1.72 ± 0.61	1.85 ± 0.79	1.77 ± 0.44
**CCR2**	-1.48 ± 0.62	-0.15 ± 0.68	0.75 ± 0.21	0.89 ± 0.29	0.79 ± 0.24
**CX3CR1**	-1.56 ± 0.16	-0.97 ± 0.83	0.14 ± 0.14	0.13 ± 1.03	-0.52 ± 0.95
**CCR7**	-1.11 ± 1.35	-0.63 ± 0.70	1.37 ± 0.66	2.15 ± 1.11	1.63 ± 1.04
**CXCR2**	-1.20 ± 0.03	-0.74 ± 0.73	-0.26 ± 0.51	0.12 ± 0.81	0.24 ± 0.74
**CCL2**	-1.55 ± 0.59	-0.52 ± 0.67	2.24 ± 0.92	2.06 ± 0.76	1.88 ± 0.87
**CCL3**	-1.79 ± 0.58	-0.39 ± 0.91	1.63 ± 0.69	1.90 ± 0.57	1.65 ± 0.60
**CCL4**	-1.38 ± 0.91	-0.19 ± 1.10	2.66 ± 1.24	2.97 ± 1.28	2.64 ± 1.20
**CCL5**	-1.40 ± 0.71	-0.13 ± 1.03	3.58 ± 1.48	4.00 ± 1.78	4.05 ± 1.91
**CCL7**	-1.26 ± 0.41	0.00 ± 0.23	1.69 ± 0.16	2.07 ± 0.65	1.84 ± 0.26
**CCL20**	-1.38 ± 0.46	-0.91 ± 0.58	-0.22 ± 1.17	0.07 ± 1.29	-0.10 ± 0.83
**CXCL1**	-3.31 ± 1.57	-1.67 ± 0.91	-0.38 ± 1.54	-0.12 ± 1.44	-0.38 ± 1.56
**CXCL9**	-1.04 ± 0.67	-0.91 ± 0.95	4.36 ± 0.81	4.30 ± 0.81	4.32 ± 0.64
**FOXP3**	-1.85 ± 0.74	-0.20 ± 1.07	1.49 ± 0.90	1.85 ± 0.68	1.29 ± 0.82
**TBX21**	-1.99 ± 0.99	0.00 ± 1.52	1.32 ± 0.77	1.52 ± 0.33	0.92 ± 0.60
**RORC**	-0.41 ± 0.71	0.14 ± 0.90	0.40 ± 0.34	0.64 ± 0.36	0.24 ± 0.35
**ARG1**	-0.26 ± 1.43	0.16 ± 1.25	2.87 ± 1.31	2.59 ± 1.00	3.09 ± 1.41
**NOS2**	-1.34 ± 0.99	0.54 ± 1.56	4.15 ± 1.58	4.28 ± 1.42	3.86 ± 1.43
**IL-17A**	-3.29 ± 0.43	-4.82 ± 3.66	-0.26 ± 0.05	-5.91 ± 6.89	-5.84 ± 6.26
**IL-17F**	4.05 ± 6.66	-1.04 ± 1.31	4.93 ± 4.49	1.96 ± 0.82	-1.02 ± 0.89
**IL-17D**	-0.67 ± 1.34	-0.65 ± 0.85	0.18 ± 0.20	0.51 ± 0.43	0.04 ± 0.11
**TNFα**	-0.61 ± 0.98	-0.73 ± 0.55	1.94 ± 0.79	2.25 ± 1.18	2.04 ± 2.59
**IL-12a**	-1.11 ± 1.13	-0.29 ± 1.43	4.12 ± 2.80	4.38 ± 2.29	4.19 ± 2.59
**IL-6**	-1.58 ± 1.65	-1.86 ± 1.08	0.24 ± 0.78	0.71 ± 0.66	0.05 ± 0.84
**IFNβ**	-1.34 ± 0.40	-0.90 ± 0.80	-0.20 ± 0.23	0.29 ± 0.75	-0.23 ± 0.52
**IFNγ**	-2.59 ± 0.95	0.21 ± 1.36	2.46 ± 1.07	3.07 ± 0.77	2.15 ± 0.92
**IL-10**	-4.21 ± 2.04	-1.48 ± 0.64	-0.49 ± 1.12	0.26 ± 1.27	-0.98 ± 1.21

**IL-4**	-2.08 ± 0.33	6.28 ± 7.16	5.99 ± 5.69	6.88 ± 5.19	6.13 ± 5.50
**IL-13**	-2.10 ± 1.08	-1.13 ± 0.95	-0.30 ± 1.28	0.52 ± 0.72	-0.38 ± 0.98
**IL-33**	-0.64 ± 0.34	-0.36 ± 0.45	0.17 ± 0.25	0.08 ± 0.52	0.13 ± 0.22
**TSLP**	-0.62 ± 1.23	0.91 ± 1.36	2.36 ± 1.31	1.90 ± 0.57	0.84 ± 0.36

Table S1. Inflammatory gene expression between *S. aureus, L. major,* and *L. major-S. aureus* coinfected ears at different doses at 28 days post-infection. C_T values were normalized to GAPDH and to the average value of the PBS group for each assay to get the $-\Delta\Delta C_T$, yielding the $\log_2$(fold change). Data shown as the mean ± SEM of three independent experiments, each with 4-5 mice/group.
